# T1 and FLAIR signal intensities are related to tau pathology in dominantly inherited Alzheimer disease

**DOI:** 10.1002/hbm.26514

**Published:** 2023-10-23

**Authors:** Farzaneh Rahmani, Matthew R. Brier, Brian A. Gordon, Nicole McKay, Shaney Flores, Sarah Keefe, Russ Hornbeck, Beau Ances, Nelly Joseph‐Mathurin, Chengjie Xiong, Guoqiao Wang, Cyrus A. Raji, Jorge J. Libre‐Guerra, Richard J. Perrin, Eric McDade, Alisha Daniels, Celeste Karch, Gregory S. Day, Adam M. Brickman, Michael Fulham, Clifford R. Jack, Christian la La Fougère, Gerald Reischl, Peter R. Schofield, Hwamee Oh, Johannes Levin, Jonathan Vöglein, David M. Cash, Igor Yakushev, Takeshi Ikeuchi, William E. Klunk, John C. Morris, Randall J. Bateman, Tammie L. S. Benzinger, For the Dominantly Inherited Alzheimer Network (DIAN)

**Affiliations:** ^1^ Washington University School of Medicine St. Louis Missouri USA; ^2^ Mayo Clinic, Department of Neurology Jacksonville Florida USA; ^3^ Taub Institute for Research on Alzheimer's Disease & the Aging Brain, and Department of Neurology College of Physicians and Surgeons Columbia University New York New York USA; ^4^ Royal Prince Alfred Hospital (RPA) Sydney Australia; ^5^ Mayo Clinic Rochester Minnesota USA; ^6^ Department of Nuclear Medicine and Clinical Molecular Imaging University Hospital Tuebingen Tübingen Germany; ^7^ German Center for Neurodegenerative Diseases (DZNE) Tuebingen Tübingen Germany; ^8^ Department of Preclinical Imaging and Radiopharmacy Eberhard Karls University Tübingen Tübingen Germany; ^9^ Neuroscience Research Australia Sydney New South Wales Australia; ^10^ School of Biomedical Sciences University of New South Wales Sydney New South Wales Australia; ^11^ Brown University Providence Rhode Island USA; ^12^ Department of Neurology Ludwig‐Maximilians‐Universität München Munich Germany; ^13^ German Center for Neurodegenerative Diseases (DZNE), site Munich Munich Germany; ^14^ Munich Cluster for Systems Neurology (SyNergy) Munich Germany; ^15^ UK Dementia Research Institute at University College London London UK; ^16^ Dementia Research Centre UCL Queen Square Institute of Neurology London UK; ^17^ Niigata University Brain Research Institute Niigata Japan; ^18^ University of Pittsburgh Pittsburgh Pennsylvania USA

**Keywords:** amyloid PET, dominantly inherited Alzheimer disease, quantitative MR imaging, T1 and FLAIR signal intensity, tau PET

## Abstract

Carriers of mutations responsible for dominantly inherited Alzheimer disease provide a unique opportunity to study potential imaging biomarkers. Biomarkers based on routinely acquired clinical MR images, could supplement the extant invasive or logistically challenging) biomarker studies. We used 1104 longitudinal MR, 324 amyloid beta, and 87 tau positron emission tomography imaging sessions from 525 participants enrolled in the Dominantly Inherited Alzheimer Network Observational Study to extract novel imaging metrics representing the mean (*μ*) and standard deviation (*σ*) of standardized image intensities of T1‐weighted and Fluid attenuated inversion recovery (FLAIR) MR scans. There was an exponential decrease in FLAIR‐μ in mutation carriers and an increase in FLAIR and T1 signal heterogeneity (T1‐*σ* and FLAIR‐*σ*) as participants approached the symptom onset in both supramarginal, the right postcentral and right superior temporal gyri as well as both caudate nuclei, putamina, thalami, and amygdalae. After controlling for the effect of regional atrophy, FLAIR‐*μ* decreased and T1‐*σ* and FLAIR‐*σ* increased with increasing amyloid beta and tau deposition in numerous cortical regions. In symptomatic mutation carriers and independent of the effect of regional atrophy, tau pathology demonstrated a stronger relationship with image intensity metrics, compared with amyloid pathology. We propose novel MR imaging intensity‐based metrics using standard clinical T1 and FLAIR images which strongly associates with the progression of pathology in dominantly inherited Alzheimer disease. We suggest that tau pathology may be a key driver of the observed changes in this cohort of patients.

## Graphical abstract

Orange and blue brain regions respectively show regions with a significant increase or decrease in our new MR intensity‐based biomarkers (T1 Mu, T1 sigma, FLAIR mu and FLAIR sigma) or the conventional PET imaging biomarkers (Tau PET and Amyloid PET), when compared between mutation carriers versus  non‐carriers of dominantly‐inherited Alzheimer disease. Great intensity of color corresponds to a smaller p‐value of the tested model, while EYO corresponds to accumulation of pathology over the years prior to symptom onset. Note that the temporal and regional distribution of abnormalities in FLAIR Mu, our most important intensity‐based biomarker, closely resemble those of tau PET.

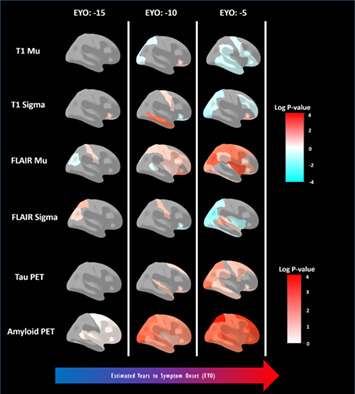

**Abbreviations**
AV1451: [^18^F]AV‐1451 (flortaucipir)
CDR: Clinical Dementia Rating scale
FDR: false‐discovery rate
PiB: ^11^C Pittsburgh Compound B
SUVR: standard uptake value ratio
*μ*
: mean/average
*σ*
: standard deviation

## 1 INTRODUCTION

Studies of imaging‐based biomarkers demonstrate the existence of a preclinical phase of Alzheimer disease wherein pathological changes accumulate in the absence of overt clinical symptoms (Jack et al., 2013; Morris & Price, 2001). Accurate identification of this preclinical period is critical to Alzheimer disease treatment that require early, pre‐symptomatic intervention (Bateman et al., 2011; Bateman et al., 2017; Grill et al., 2020; Sperling et al., 2014). Since brain MRI is part of the standard care of progressive cognitive decline, biomarkers measurable on routinely acquired clinical MR images that track pathological changes could supplement the extant invasive (cerebrospinal fluid analysis), logistically challenging and expensive (e.g., amyloid or tau positron emission tomography [PET]), or yet to be clinically validated blood‐based biomarkers (Jack et al., 2010, 2012; Knopman et al., 2001; Li et al., 2022; Zetterberg & Bendlin, 2021). Among these, the metrics directly computed from MR voxel intensities could complement the information available from routine MR images by enabling fast and early detection of pathologies or existing histology with little to no added computational or resource cost (Glasser & van Essen, 2011a; Keenan et al., 2019; Koenig et al., 2021). We have developed a new set of semi‐quantitative structural biomarkers based on standardization of voxel intensity distribution of T1‐weighted (T1w) and fluid‐attenuated inversion recovery (FLAIR) images that have shown promise in predicting disability in multiple sclerosis (Brier et al., 2021). Our aim, using routine clinical T1w and FLAIR MR imaging, was to investigate the utility of this technique in pre‐clinical and clinical patients with dominantly inherited Alzheimer disease.

The Dominantly Inherited Alzheimer Network Observational Study (DIAN‐Obs) cohort is an international research partnership where the aim is to study the clinical and imaging course of individuals who carry one of the gene mutations responsible for dominantly inherited Alzheimer disease (Bateman et al., 2012). These individuals develop Alzheimer disease with a predictable age‐at‐symptomatic onset as early as 30–50 years of age (Bateman et al., 2012). Asymptomatic carriers of disease‐causing mutations represent a unique opportunity to study novel imaging biomarkers relevant to the preclinical phase of the disease. In addition, novel findings from dominantly inherited Alzheimer disease are also applicable to the more common, late‐onset form of Alzheimer disease (Benzinger et al., 2013; Gordon et al., 2019; Lee et al., 2016; McDade et al., 2018).

Our aims, using this new approach on MR scans within the DIAN‐Obs cohort were to: (1) identify differences in the T1w and FLAIR cortical intensity distribution in individuals with dominantly inherited Alzheimer disease‐causing mutations versus non‐carrier controls; (2) explore changes in the T1w and FLAIR cortical intensity distribution in individuals with these mutations along the disease trajectory from the asymptomatic to symptomatic stage; and (3) identify any relationship between these MR signal changes and amyloid and tau pathology as measured by amyloid PET and tau PET, respectively.

## 2 METHODS

### 2.1 Participants

Participants were enrolled from the fifteenth semiannual data freeze of a cohort of individuals recruited by the DIAN‐Obs study (https://dian.wustl.edu/our-research/observational-study/). DIAN‐Obs participants were recruited since 2009 by 22 different sites on the basis of being a child of a person with a known dominantly inherited Alzheimer disease‐causing mutation; including *PSEN1*, *PSEN2*, and *APP*. Clinical, imaging, and genetic data were included from all participants that passed the quality control (525 participants, 1104 MR, 324 amyloid beta PET, and 87 tau PET imaging sessions). Participants from families with the Dutch *APP* E693Q mutation were excluded as this mutation is responsible for hereditary cerebral hemorrhage with amyloidosis (Bugiani et al., 2010) and blood products distort the MR signal and would thus potentially interfere with the planned analyses. Each participant's first clinical assessment was considered as the reference baseline. For asymptomatic participants, imaging and clinical testing are performed every 2 years until they are within 5 years of their expected age of symptom onset (AO) based on their parental (if mutation status is unknown) or mean mutation (for mutation carriers) AO. Thereafter, or once cognitive symptoms developed, assessments are performed annually. Amyloid beta PET and tau PET scans were included if they were acquired within 18 months of an MR imaging session.

Included studies were all collected between January 2009 and June 2020 and the corresponding T1w and FLAIR, amyloid beta PET, and tau PET images were used for subsequent analyses. The Institutional Review Board at Washington University in St. Louis provided supervisory review and human studies approval, as did the regulatory committees at each of the participating institutions. Participants provided written informed consent in accordance with their local institutional review board.

### 2.2 Clinical assessments

Global Clinical Dementia Rating® scale (CDR®) evaluation was performed at every clinical visit by an experienced clinician (Morris, 1993). At each clinical visit estimated years from symptom onset (EYO) were calculated by subtracting the participant's age on that visit from the predicted AO. The AO was defined as: (1) participants own AO if they were symptomatic; (2) average AO reported across individuals with the same specific mutation in asymptomatic mutation carriers; or (3) parental AO if mutation status was unknown (Ryman et al., 2014).

### 2.3 Image processing and analysis

#### 2.3.1 T1 and FLAIR image acquisition and processing

Structural MR images (T1w and FLAIR) were acquired on 3 Tesla Siemens or Philips scanners using a protocol designed to match the ADNI‐2 MRI protocol (Jack et al., 2008). The T1w magnetized prepared rapidly acquired gradient echo and the axial FLAIR images were acquired with protocols previously described (McKay et al., 2022).

### 2.4 Cortical thickness and volume

T1w images were processed with FreeSurfer 5.3 and resampled to 1 × 1 × 1 mm resolution for volumetric segmentation and cortical reconstruction (Fischl, 2012). Thickness and grey matter volumes for 68 cortical regions and volumes for 12 subcortical grey matter structures in left and right hemispheres were derived after quality control of FreeSurfer output through visual inspection and manual editing of the cortical and subcortical segmentation output when necessary (https://surfer.nmr.mgh.harvard.edu/fswiki/LongitudinalEdits).

### 2.5 T1w and FLAIR image intensity normalization

Among available structural MR sequences, T1 and FLAIR sequences were selected due to their established ability to characterize brain structure and white matter pathology as part of the initial work up of patient with dementia (Knopman et al., 2001). While T1w and FLAIR image intensities are proportional to T1 and T2, respectively, they are expressed on an arbitrary intensity scale. This scale differs across individual participants, acquisition parameters, and scanners. We adopted an intensity normalization strategy that minimizes these differences and allows for the semi‐quantitative comparison of image intensity distribution properties (Brier et al., 2021). This is accomplished by registering the bispectral intensity distribution (T1w, FLAIR) to a normative intensity atlas. In brief, a normative reference was created from a sample 101 healthy individuals imaged using similar high‐resolution imaging acquisition parameters as published previously (Brier et al., 2021). After brain extraction and field inhomogeneity correction (Smith, 2002; Zhang et al., 2001), a bivariate histogram, with T1w/FLAIR voxel intensities represented on the axes, was created for each participant. Next, individual histograms were affine‐registered to the reference histogram to achieve intensity normalization. This intensity transformation was applied to each participant's T1w and FLAIR data, generating intensity‐normalized images. Finally, regional T1w and FLAIR intensities were extracted for 68 cortical and 12 subcortical grey matter structures of interest based on FreeSurfer. All further analyses were performed using the mean (*μ*) and standard deviation (*σ*) of the T1 and FLAIR signals, T1‐*μ*, T1‐*σ*, FLAIR‐*μ*, and FLAIR‐*σ*, within each region, collectively referred to as image intensity metrics. All available MR scans from all participants were suitable for analyses.

### 2.6 Amyloid beta PET acquisition and processing

Amyloid beta PET scans were matched to their respective MR imaging session within 12 months of acquisition (*μ* ± *σ* time difference: 15.3 ± 34.7 days). Estimation of amyloid deposition was achieved through injection of 8–18 mCi of ^11^C Pittsburgh Compound B ([^11^C]PiB) followed by PET imaging using a modified version of the ADNI PET acquisition protocol (McKay et al., 2022; Mueller et al., 2005). Attenuation correction was performed using a separate CT scan obtained before the PET (Gordon et al., 2019). PET data were analyzed using the FreeSurfer‐based PET Unified Pipeline (Rousset et al., 2008; Su et al., 2013; Su et al., 2015). Data from the 40–70 min postinjection window were used to generate the partial volume corrected regional standard uptake value ratio (SUVR, cerebellar grey matter reference region), within each of 68 cortical and 12 subcortical FreeSurfer‐defined grey matter regions‐of‐interest in both hemispheres (Su et al., 2018).

### 2.7 Tau PET acquisition and processing

Tau PET scans were matched to their respective MR imaging sessions performed with an 18‐month interval (*μ* ± *σ* time difference: 181 ± 33.3 days). Tau PET imaging was performed through injection of 8–11 mCi of the [^18^F]AV‐1451 (flortaucipir; Eli Lilly and Company, Indianapolis, IN) tracer following the standard DIAN protocol as described before (Gordon et al., 2019; McKay et al., 2022). Regional tau SUVR values (cerebellar grey matter reference region) were generated for 68 cortical and 12 subcortical FreeSurfer‐defined grey matter regions‐of‐interest in both hemispheres using the 40–60 min post‐injection window of each scan.

### 2.8 Statistical analyses

Statistical analyses were performed using the R software version 4.0.5. We used the *shapiro.test* function from the stats package to investigate the normality assumption for the residuals of the demographic variables as well as the image intensity metrics. To compare the demographic variables and image intensity metrics between mutation carriers and non‐carriers, we used a Welch two‐sample *t*‐test, or the Wilcoxon signed‐rank test if the normality assumption was not met. Next, the *lmer* function from the package *lme4* (Bates et al., 2015) was used to investigate the interactive effect between *EYO* and mutation carrier status on regional image intensity metrics using either of the models below (Model 1: baseline MR session and Model 2: longitudinal MR sessions):

Model 1 (using baseline MR imaging sessions only):

(1) $$\mathrm{IIM}\sim \mathrm{EYO}\times \text{Mutation}+\text{Regional thickness or volume}+\left(1|\text{Family}\;\mathrm{ID}\right)$$

Model 2 (using all MR imaging sessions):

(2) $$\begin{array}{l} \mathrm{IIM}\sim \text{Time}\times \text{Baselin}e_{\mathrm{EYO}}*\text{Mutation}+\text{Time}\\ \;*\;\text{Regional thickness or volume}+\left(1|\text{Family}\;\mathrm{ID}\right)+\left(1|\text{Subject}\;\mathrm{ID}\right). \end{array}$$

Regional cortical thickness or subcortical volumes were added as a fixed factor to all prediction models to account for effects of non‐interest, for example, concurrent atrophy in cortical and subcortical regions. Since the interaction of regional thickness and volumes with mutation status and their changes over *EYO* have been extensively studied in the DIAN‐Obs cohort, we did not include those terms in our models (Bateman et al., 2012; Dincer et al., 2020; Weston et al., 2016). Furthermore, the family identification number (the specific family from which the individual was recruited) was added as random factor to all prediction models (1|Family_ID), and the individual identification number was added as random factor to all models using longitudinal sessions (1|Subject_ID). In models using all MR image sessions (Models 2 and 4: longitudinal data points), participants' *EYO* at the time of recruitment MR scan was considered as Baseline *EYO*. In these models the *Time* variable was calculated as the time difference between each MR imaging session date and the date of the first MR scan. *P‐value*s of all models were corrected for multiple comparisons using the Benjamini–Hochberg false‐discovery rate (FDR) correction (Benjamini & Hochberg, 1995). An FDR‐corrected *P‐value* of below .05 in the *EYO* × *Mutation* (Model 1) or *Time* × *EYO* × *Mutation* (Model 2) terms, was used to identify significant first‐degree interactive effects between *EYO* and *mutation* status in predicting image intensity metrics in each cortical region.

In the next set of models, we investigated whether the addition of the interactive effect between the quadratic *EYO* term (*EYO* ^2^) and mutation status would improve the model fit (Model 3: baseline MR session and Model 4: longitudinal MR sessions):

Model 3 (using baseline MR imaging sessions only):

(3) $$\begin{array}{l} \mathrm{IIM}\sim \mathrm{EYO}\times \text{Mutation}+({\mathrm{EYO}}^{2})\times \text{Mutation}\\ \;+\;\text{Regional thickness or volume}+\left(1|\text{Family}\;\mathrm{ID}\right) \end{array}$$

Model 4 (using all MR imaging sessions):

(4) $$\begin{array}{l} \mathrm{IIM}\sim \text{Time}\times {\text{Baseline}}_{\mathrm{EYO}}\times \text{Mutation}+\text{Time}\;\times \left(\text{Baselin}{e_{\mathrm{EYO}}}^{2}\right)\\ \;\times \;\text{Mutation}+\text{Time}\times \text{Regional thickness or volume}+\left(1|\text{Family}\;\mathrm{ID}\right)\\ \;+\;\left(1|\text{Subject}\;\mathrm{ID}\right). \end{array}$$

We compared the fits of the two sets of models (with and without the *EYO* ^2^) using the *ANOVA* function between each pair of models (Model 1 vs. Model 3 and Model 2 vs. Model 4; Rouder et al., 2016). If the interaction between *EYO* × *Mutation* was significant from the first degree model (Model 1 and Model 2), an ANOVA FDR‐corrected *P‐value* of below 0.05, in the presence of a significant $\left(\text{Baseline}\_{\mathrm{EYO}}^{2}\right)\times \text{Mutation}$ interaction, signified an improvement in the model fit with addition of the quadratic terms of *EYO*.

Finally, we investigated the relationship between regional image intensity metrics and amyloid and tau pathology through a partial correlation test using the *pcor.test* function in R from the ppcor package. Similar to the previous models, cortical thicknesses or subcortical volumes were added as a covariate to both correlation models. Each partial correlation model was then replicated in mutation carriers (*n* = 313) and, separately, in symptomatic (i.e., global *CDR* > 0) mutation carriers (*n* = 102) separately in order to investigate the modifying effect of mutation carrier and cognitive status on each relationship.

## 3 RESULTS

Table 1 summarizes the baseline clinical, demographic and imaging biomarkers of *Mutation* carrier and non‐carrier groups. Mutation carrier and non‐carriers were comparable in age, sex, race composition, and apolipoprotein‐E4 allele carrier status. As expected, *Mutation* carriers had higher global CDR scores compared with non‐carriers. Table 2 shows group differences in image intensity metrics between Mutation carriers and non‐carriers. Table S1 demonstrates the number of imaging sessions, participants, average, and cumulative follow‐up times of participants contributing to each set of longitudinal sessions (MR, Tau PET, and amyloid beta PET).

**TABLE 1:** Clinical, demographic, and imaging biomarkers of participant groups in the baseline.

	Total (*n* = 517)	Mutation non‐carriers (*n* = 204)	Mutation carriers (*n* = 313)	*P*‐value^a^
Age, years; median (Q1–Q3)^b^	36 (30–45)	36 (30–44)	36 (30–44)	.566
Sex				
Men, *n* (%)	225 (43.5%)	86 (42.2%)	139 (44.4%)	.614
Women, *n* (%)	292 (56.4%)	118 (57.8%)	174 (55.6%)	
Race				
Caucasian, *n* (%)	448 (86.7%)	182 (89.3%)	272 (86.9%)	.393
Asian, *n* (%)	20 (3.9%)	6 (2.9%)	14 (4.5%)	
African American, *n* (%)	2 (0.4%)	0 (0%)	2 (0.6%)	
Native Hawaiian or Pacific Islander, *n* (%)	4 (0.8)	1 (0.5%)	3 (1%)	
Others, *n* (%)	37 (7.2%)	15 (7.3%)	22 (7%)	
Education, years; median (Q1–Q3)^b^	14 (12–16)	15 (13–16)	14 (12–16)	.296
DIAN‐EYO at baseline, years; median (Q1–Q3)^b^	−9.4 (−18.6 to 0.46)	−11 (−19 to −1.3)	−7.8 (−18.2 to 1.4)	**.019**
Mutation type				
PSEN1, *n* (%)	368 (71.2%)	138 (67.6%)	230 (73.5%)	
PSEN2, *n* (%)	43 (8.3%)	19 (9.4%)	24 (76.6%)	.359
APP, *n* (%)	106 (20.5%)	47 (23%)	59 (18.9%)	
Dutch mutation carrier, *n* (%)	19 (3.7%)	10 (4.9%)	9 (2.9%)	.172
APOE‐ε4 carrier, *n* (%)	153 (29.5%)	62 (30%)	91 (29%)	.382
CDR global score				
0, *n* (%)	401 (77.5%)	190 (93.1%)	211 (67.45)	
0.5, *n* (%)	80 (15.4%)	14 (6.9%)	66 (21%)	
1, *n* (%)	27 (5.2%)	0	27 (8.6%)	**<.001**
2, *n* (%)	7 (1.4%)	0	7 (2.2%)	
3, *n* (%)	2 (0.3%)	0	2 (0.6%)	

Abbreviations: APOE‐ε4, apolipoprotein epsilon 4 allele; APP, Amyloid Beta precursor protein gene; CDR, Clinical Dementia Rating Scale; DIAN‐EYO, estimated years to symptom onset based on a combination of symptomatic participants age of onset, mean mutation age of onset, and parental age of onset; Dutch mutation, E693Q mutation in the APP gene responsible for hereditary cerebral hemorrhage with amyloidosis; PSEN1, Presenilin 1 gene; PSEN2, Presenilin 2.

^a^ Mann–Whitney *U* test was used to compare the distribution of continuous variables across groups. Pearson's Chi‐square test was used to compare frequencies of all other variables between mutation carrier and non‐carrier groups. Bold values indicate test with statistical significance considering a threshold of .05.

^b^ None of the continuous variables had a normal distribution and are therefore reported as median plus the first and third quartiles (Q1–Q3). Normality was determined using the Kolmogrov‐Smirnov Goodness‐of‐Fit test.

**TABLE 2:** Comparing image intensity metrics in baseline regions between mutation carrier and non‐carrier participants.

Region	Hemi	T1‐*μ P*‐value^a^	T1‐*σ P*‐value^a^	FLAIR‐*μ P*‐value^a^	FLAIR‐*σ P*‐value^a^	T1‐*μ* mean diff^b^	T1‐*σ* mean diff^b^	FLAIR‐*μ* mean diff^b^	FLAIR‐*σ* mean diff^b^
Inferior parietal	Left	.38	***<.001***	**.04**	**.002**	2.97	10.68	−12.34	15.15
Lateral occipital	Left	.13	***.005***	.64	.52	6.85	7.54	−3.99	4.68
Lingual	Left	.39	***.048***	.37	.87	3.52	3.50	−6.21	0.90
Pars orbitalis	Left	.94	***.006***	.58	.28	0.45	13.90	−6.56	8.35
Pars triangularis	Left	.64	***.004***	***.049***	.14	1.79	10.45	−12.98	8.31
Pericalcarine	Left	.17	***.033***	.64	.12	5.77	3.91	−2.82	6.12
Postcentral	Left	.22	***.004***	.083	.12	4.43	9.65	−9.75	8.44
Rostral middle frontal	Left	.19	***<.001***	**.011**	.063	−6.30	19.92	−18.13	14.74
Superior frontal	Left	.13	***.037***	.59	.72	6.19	6.49	−3.55	2.07
Superior parietal	Left	.17	***.006***	***.049***	.12	4.98	9.14	−10.74	9.26
Supramarginal	Left	.28	***.004***	***.049***	***.004***	3.46	7.50	−10.40	11.51
Inferior parietal	Right	.21	***.001***	***.15***	***.004***	4.31	7.70	−8.21	12.37
Pars orbitalis	Right	.64	***.037***	.64	.36	2.67	11.58	−4.39	7.17
Pars triangularis	Right	.39	***.001***	.25	**.019**	3.28	11.22	−7.75	11.67
Postcentral	Right	.34	***.002***	***.049***	**.019**	3.47	10.13	−12.36	11.08
Rostral middle frontal	Right	.55	***.003***	***.049***	.14	−3.00	15.43	−13.70	12.55
Superior parietal	Right	.13	***.006***	.36	.12	5.71	7.39	−5.37	7.98
Pars opercularis	Left	.19	.1	***.049***	.25	4.46	3.99	−11.22	5.27
Precuneus	Left	.13	.07	***.049***	.23	5.60	3.97	−10.51	4.58
Transverse temporal	Left	.43	.38	***.049***	.52	3.02	1.60	−13.25	2.89
Precuneus	Right	.15	.1	***.049***	.33	4.87	3.36	−11.48	3.83
Supramarginal	Right	.13	.08	***.049***	**.025**	5.20	4.13	−10.40	8.48
Frontal pole	Right	.12	.1	***.049***	.87	−28.09	15.42	−35.14	2.59
Transverse temporal	Right	.61	.15	***.003***	.14	1.75	2.89	−19.62	6.17
Amygdala	Left	.20	.91	.087	***.004***	−4.8	−0.01	2.1	−1.4
Caudate	Left	.20	.42	***.006***	.44	−5.7	0.54	5.6	1.17
Hippocampus	Left	.84	.21	.11	***.004***	−0.03	−0.73	0.75	−5.1
Pallidum	Left	.88	.26	***.08***	.44	−1.3	0.18	−3	1.04
Putamen	Left	.20	***<.001***	***.025***	.47	−5.2	1.57	2.7	0.1
Thalamus	Left	.99	.30	***<.001***	***.004***	−2.3	0.7	5.8	−4.18
Amygdala	Right	.15	.30	***.001***	.44	0.47	−0.24	−2.8	−2.3
Caudate	Right	.20	.36	***.011***	.44	−2.7	−0.03	3.2	1.8
Hippocampus	Right	.84	.39	.24	.44	−2.4	0.21	4.7	−2.4
Pallidum	Right	.80	.40	***.008***	.67	−6.8	1.7	6.3	−0.47
Putamen	Right	.15	***.008***	***.007***	.81	−4.1	0.5	5.2	−0.5
Thalamus	Right	.91	.30	***<.001***	***.045***	−2.3	−1.1	−0.06	−2.4

Abbreviations: hemi, hemisphere; mean diff: mean difference.

^a^ *P*‐values are corrected for false discovery rate using the Benjamini–Hochberg method.

^b^ Mean different in image intensity metrics by subtracting the group average in mutation carriers from than in non‐carriers.

### 3.1 Cortical image intensity depends on years to disease onset and mutation status

**FIGURE 1:**
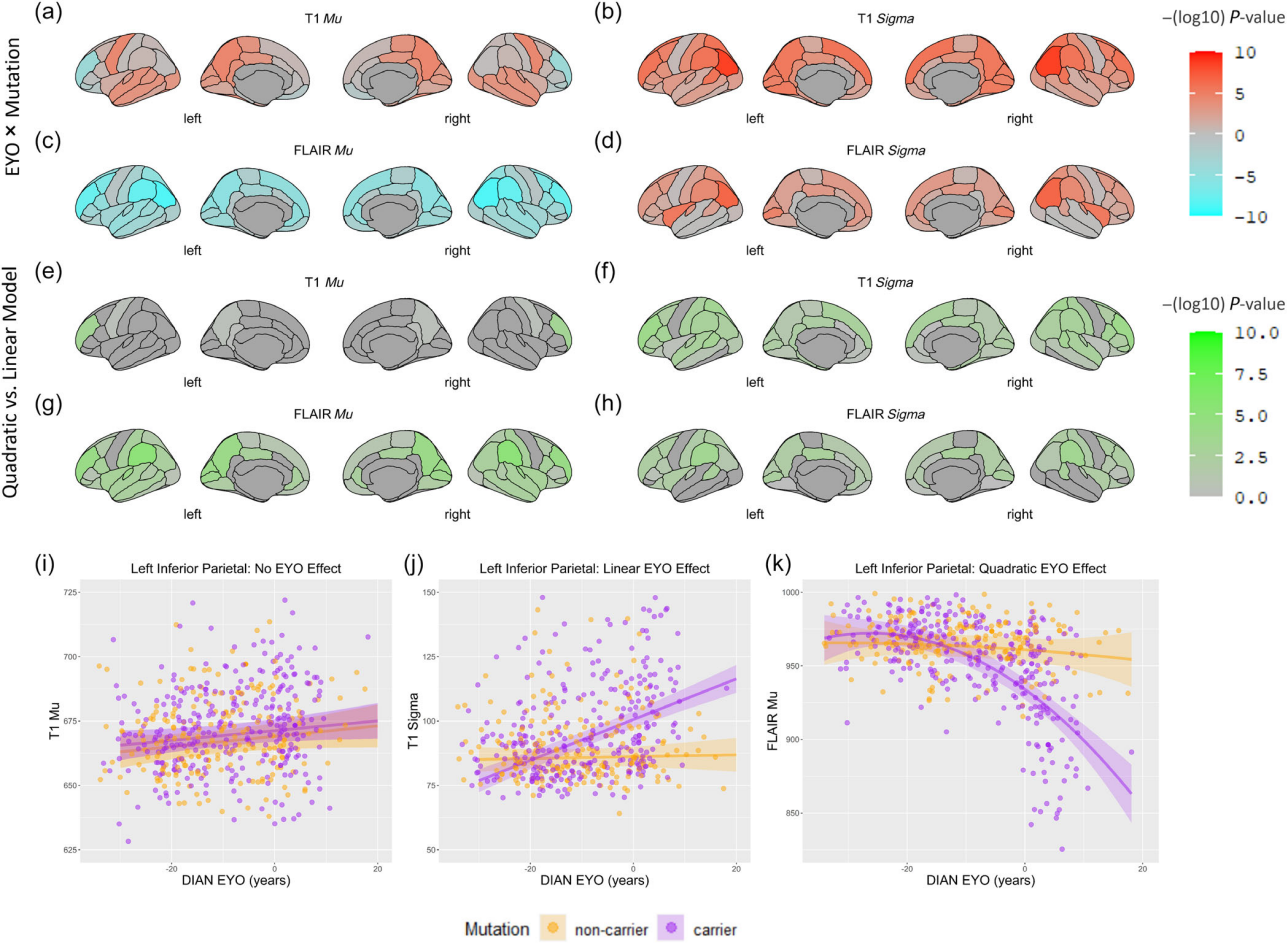
Cortical regions with a significant interaction between EYO and mutation carrier status in relationship to image intensity metrics in the baseline cohort. Panels (a) through (d) demonstrate the negative log10 of *P‐value* for the interaction term between EYO and mutation carrier status in the linear model predicting image intensity metrics. This value is multiplied by the sign of the beta coefficient of the interaction term, so that areas in which mutation carriers demonstrated increasingly lower image intensity metrics with advancing EYO are coded as Cyan and areas with increasingly higher image intensity metrics with advancing EYO are coded as Orange. The colors intensities are inversely proportional to the *P‐value* of the interaction. Panels (e) through (h) demonstrate the negative log10 of *P‐value* for the ANOVA test comparing the models with (Model 3) and without (Model 1) the quadratic term of EYO^2. As a result, areas in which the addition of the quadratic EYO^2^ term improved the prediction of image intensity metrics are shown with different intensities of Green. Panel (i) is example of a region where there was no interaction between mutation carrier status and EYO in predicting T1‐*μ*. Panel (j) is example of a region with a significant linear interaction between EYO and mutation carrier status and no improvement in the model with the addition of the EYO^2^ term in predicting T1‐*σ*. Panel (k) is example of a region with a significant improvement in model prediction with the addition of the EYO^2^ term over the linear term in predicting FLAIR‐*μ*. EYO, estimated years to onset of symptoms; image intensity metrics, image intensity metric based on mean or standard deviation of the intensity in the cortical regions in the T1 or FLAIR images (T1‐*μ*, T1‐*σ*, FLAIR‐*μ*, and FLAIR‐*σ*).

Effects of interest are image intensity abnormalities that increase in severity with advancing *EYO* in *Mutation* carrier group. Statistically, this manifests as a relationship between image intensity metrics and an interaction between *EYO* and *Mutation* status. In the cross‐sectional data (Model 1: baseline MR sessions), a subset of cortical regions demonstrated higher *T1‐σ*, *FLAIR‐σ*, and *T1‐μ* and lower *FLAIR‐μ* in *Mutation* carriers as these individuals approach onset of Alzheimer disease (Figure 1a–d). While changes in *T1‐σ*, *FLAIR‐σ*, and *FLAIR‐μ* were widespread, changes in *T1‐μ* were limited to the medial parietal lobe regions. FLAIR hypointensity and increased T1 and FLAIR signal heterogeneity effects were prominent in regions previously noted to demonstrate atrophy (Dincer et al., 2020) but also in additional areas such as the left inferior and superior parietal and right lateral occipital lobes. As we have already accounted for the effect of atrophy through addition of the regional thickness or volume to all model terms, we attribute these changes to underlying dominantly inherited Alzheimer disease pathology.

Visual inspection of the regression fit line in regions with a significant *EYO* × *Mutation* interactions suggested that a quadratic fit may be more appropriate in some regions. To compare the fit of models with addition of *EYO* ^2^ terms, we refit the model with the addition of a quadratic *EYO* term (*EYO* ^2^) and its interaction with *Mutation* (Model 3). The degree to which the quadratic terms improve the model fits are represented in Figure 1e–h as the negative log_10_ of the *P‐value* resulting from an ANOVA test between Model 1 (linear effect of *EYO* only) and Model 3 (addition of *EYO* ^2^ terms). Addition of the quadratic terms resulted in better prediction of the *T1‐σ* in both middle frontal, left superior parietal and the right postcentral gyri, and also improved prediction of the *FLAIR‐σ* and *FLAIR‐μ* in the both supramarginal, right postcentral and right superior temporal gyri (Figure 1e–h). Figure 1i–k demonstrate fits lines from three different models plotted alongside the actual observations where in Figure 1i there is no significant interaction for *T1‐μ* in Figure 1j there is a significant linear *EYO* × *Mutation* interaction for T1‐σ, and in Figure 1k there is improved model fit with addition of *EYO* ^2^ terms for *FLAIR‐μ*. Cortical regions with such improvement were located at both supramarginal and inferior parietal cortices (*FLAIR‐μ*), the right posterior cingulate and right postcentral cortex (*FLAIR‐μ*), and both superior parietal cortices (*T1‐σ*).

Among subcortical regions, higher EYO was associated with decreased variability and increased average FLAIR signal in the both caudate nuclei, putamina, thalami and amygdalae, and the left hippocampus and right pallidum in mutation carriers (Tables S2–S5). Among these regions, only the left thalamus and left amygdala showed a significant quadratic relationship with EYO (Tables S2–S5). No T1 signal abnormality was seen among mutation carriers compared with their mutation negative peers with advancement of EYO.

### 3.2 Longitudinal changes in T1 and FLAIR signal are associated with progression of dominantly inherited Alzheimer disease pathology

**FIGURE 2:**
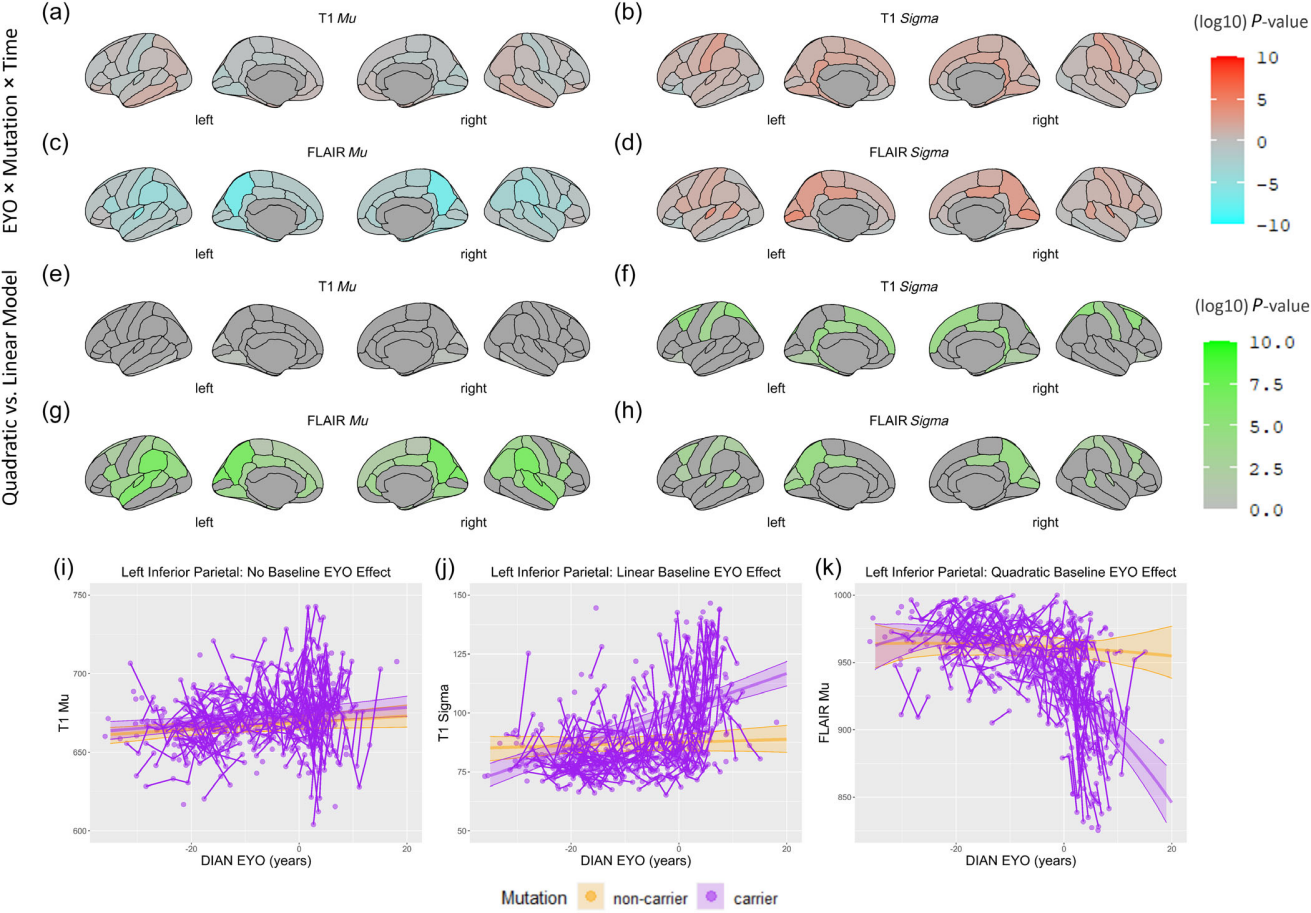
Cortical regions with a significant interaction between baseline EYO, mutation carrier status and time in relationship to image intensity metrics in the longitudinal cohort. Panels (a) through (d) demonstrate the negative log10 of *P‐value* for the interaction term between baseline EYO × mutation carrier status × time in years in the mixed effects model predicting image intensity metrics. This value is multiplied by the sign of the beta coefficient of the mentioned 3‐way interaction term, so that areas in which mutation carriers demonstrated increasingly lower image intensity metrics with advancing time are coded as Cyan and areas with increasingly higher image intensity metrics with advancing EYO are coded as Orange. The colors intensities are inversely proportional to the *P‐value* of the interaction. Panels (e) through (h) demonstrate the negative log10 of *P‐value* for the ANOVA test comparing the models with (Model 4) and without (Model 2) the quadratic term of EYO^2^. As a result, areas in which the addition of the quadratic EYO^2^ term improved the prediction of image intensity metrics are shown with different intensities of Green. Panel (i) example of a region where there was no interaction between baseline EYO and mutation carrier status over time in predicting the T1‐*μ*. Panel (j) example of a region with a significant linear interaction between baseline EYO and mutation carrier status over time, and no improvement in the model with the addition of the EYO^2^ term in predicting T1‐*σ*. Panel (j) example of a region with a significant improvement in model prediction with the addition of the EYO^2^ term over the linear term in predicting FLAIR‐*μ*. Baseline EYO, estimated years to onset of symptoms at the time of participant enrollment; image intensity metrics, image intensity metric based on mean or standard deviation of the intensity in the cortical regions in the T1 or FLAIR images (T1‐*μ*, T1‐*σ*, FLAIR‐*μ*, and FLAIR‐*σ*).

When the longitudinal data points were considered (Model 2 and Model 4), the effect of interest was the rate of change of image intensity metrics in mutation carriers with advancing time relative to the participants status in the disease trajectory at the time of recruitment (baseline EYO). Statistically, this manifests as a relationship between the image intensity metrics and an interaction between time, baseline EYO and mutation status. In these longitudinal data, this interaction term significantly predicted higher T1‐*σ*, FLAIR‐*σ*, and T1‐*μ*, and lower FLAIR‐*μ* in different cortical regions (Figure 2a–d).

As expected, Mutation carriers closer to AO had a steeper rate of change in image intensity metrics compared with carriers with higher *EYO* and non‐carriers. Similar to the results using baseline sessions, the addition of the quadratic *baseline EYO* term resulted in better model fit in prediction of *T1‐σ*, *FLAIR‐σ*, and *FLAIR‐μ* in almost all cortical regions with a significant 3‐ways interaction (Model 4 vs. Model 2) (Figure 2e–h). There was no significant change in *T1‐μ* in *Mutation* carriers during the follow up since baseline, an effect that did not change with addition of the quadratic baseline *EYO* term. The concordance between the cross‐sectional and longitudinal results suggests that these intensity metrics (*T1‐σ*, *FLAIR‐σ*, and *FLAIR‐μ*) accurately track disease progression and that *EYO* captures a significant fraction of the variance in biomarker outcomes.

When longitudinal changes in the T1 and FLAIR signal over *EYO* trajectory were studied, there was a significant interaction between *EYO* and *Mutation* status in predicting increased T1 and FLAIR signal variability in the putamina and thalami as well as left amygdala and hippocampus. Both caudate nuclei, putamina and thalami showed decreased FLAIR signal with advancing *EYO* in *Mutation* carriers (Tables S2–S5).

### 3.3 Tau pathology correlates with image intensity changes in symptomatic mutation carriers

We investigated the relationship between regional amyloid and tau pathology and image intensity metrics. For this purpose we separated participants based on *Mutation* status as a proxy of the underlying disease pathology, and based on the presence of symptoms, corresponding to the status of an individual in the disease trajectory. We therefore ran partial correlation models in: (1) Mutation carriers (Figure S1), and symptomatic *Mutation* carriers (i.e. *global CDR* > 0; Figure S2). Regional volume or thickness was considered as a covariate. Correlation coefficients for significant regions in the relationship between *PiB retention* and image intensity metrics ranged between 0.1 and 0.11 for *T1‐μ*, 0.03 and 0.1 for *T1‐σ*, and (−0.1) and (−0.03) for *FLAIR‐μ*. Correlation coefficients for significant regions in relation to regional tau uptake ranged between 0.1 and 0.24 for *T1‐σ*, (−0.3) and (−0.13) for *FLAIR‐μ*, and 0.1 and 0.24 for *FLAIR‐σ*. None of the cortical regions demonstrated significant correlation between *FLAIR‐σ* and *PiB uptake* or between *T1‐μ* and tau uptake, after FDR correction. When MR sessions of *Mutation* carriers were considered, correlation coefficients were generally higher in models correlating the amyloid burden with image intensity compared with similar models correlating tau burden and the image intensity metrics (Figure S3A–D). Nonetheless, the relationship between tau burden and these metrics was generally stronger in symptomatic *Mutation* carriers compared with similar relationships with amyloid uptake (Figure S3E–H). The stronger relationship between image intensity metrics and tau PET in symptomatic *Mutation* carriers is in line with later onset of tau pathology and its close association with symptom onset.

## 4 DISCUSSION

We used a quantitative approach applied to standard clinical T1w and FLAIR MR images to investigate changes in image‐intensity distributions across the clinical spectrum of dominantly inherited Alzheimer disease. Our main findings are that: (1) T1w and FLAIR signal variabilities are higher and the average FLAIR signal is lower as a function of disease progression in mutation carriers; (2) these MR signal changes occur, at least partly, independent from cortical atrophy and both within and outside the atrophy topography associated with dominantly inherited Alzheimer disease; and (3) closer to the AO, tau pathology was increasingly more important in driving the measured changes compared with amyloid pathology.

Quantitative MRI techniques allow for indirect assessment of intrinsic properties of brain tissue, such as myelin and iron content and synaptic density (Glasser & van Essen, 2011b; Steen et al., 2000; Zhao et al., 2017). However, the multi‐echo sequences are not routinely acquired clinically due to added scanner time (~4–5 min depending on the vendor) and limited applicability outside research settings (Callaghan et al., 2014; Knight et al., 2019; Luo et al., 2013; Wang et al., 2004; Wen et al., 2018; Zhao et al., 2017). The utility of our method lies in repurposing widely used T1w and FLAIR sequences (~1–2 min) to provide an approximation of subtle structural differences in gray matter that are associated with dominantly inherited Alzheimer disease pathology. This technique can be applied both to individual scans and in batch and once added as a tool to available image‐viewing software is expected to render results within minutes of image transfer.

Although our method relies on using two‐dimensional FLAIR images, primarily due to their widespread availability across DIAN participating sites, it can seamlessly adapt to 3D FLAIR acquisitions. The ability of our method to utilize 2D FLAIR makes it deployable across the maximum number of MRI scanners, including both 1.5 and 3 T, while 3D FLAIR requires 3 T imaging (Kakeda et al., 2012). Wide use of 3D FLAIR on 3 T scanners for specific neurological condition like Alzheimer disease is possible as this method is routinely applied for neuroimaging of persons with multiple sclerosis (Tawfik & Kamr, 2020). Notably, while the enhanced signal‐to‐noise ratio and spatial resolution provided through 3D‐FLAIR sequences can help with detection of nuanced cortical signal alterations, its benefits should be weighed against potential increases in scanner time. Modern acquisition techniques, such as compressed sensing, might address this time concern while adding to signal resolution performance of FLAIR images (Toledano‐Massiah et al., 2018).

The value of molecular imaging (PET) in the diagnosis of dementia in prodromal stages is supported by a large body of literature (Dubois et al., 2016; Luo et al., 2020). In busy clinical imaging departments factors such as cost, availability, and reimbursement limit the application of amyloid and tau PET performed in patients with preclinical AD. Different combinations of PET scanning, cerebrospinal fluid analysis and MR‐based biomarkers have been proposed to optimize patient diagnosis (Iverson et al., 2010). The American Academy of Neurology recommends the routine use of structural MRI including routine T1w and FLAIR images in the initial work up of suspected dementia (Knopman et al., 2001). While blood‐based biomarkers are rapidly developed and validated to capture pre/early symptomatic phases of AD, they are yet to be approved adapted for clinical use (Li et al., 2022). Besides the utility of T1w images in volumetric quantification of brain regions, structural MRI helps exclude anatomical or pathological abnormalities that could contribute to cognitive deficits (Knopman et al., 2001). Imaging biomarkers based on routine clinical MR sequences, such as the tool used in the current study, would therefore provide an easy‐to‐use screening tool at no added time or burden to the patients.

In recent years, however, there has substantial innovation in plasma biomarkers for neurodegenerative disease with a particular focus on Alzheimer disease. Consequently, commercially available amyloid and tau biomarkers exist for use in patients (Iaccarino et al., 2023). A key advantage of these blood tests is their specific ability to identify the amyloid and tau pathology of Alzheimer disease in accordance with the “A” and “T” respective amyloid and tau components of the current biomarker research framework (Jack et al., 2018). However, such plasma tests are not yet widely deployed while brain MRI remains a standard part of dementia evaluations and is also useful for characterizing neurodegeneration related atrophy in Alzheimer Disease (Knopman et al., 2001). In the future, both plasma and neuroimaging biomarkers of AD will be utilized in tandem to provide critical complementary data points for maximal patient benefit. Future studies will need to target the precise steps by which such information is obtained and integrated as part of this evolving clinical practice.

The amyloid and tau hypotheses of Alzheimer disease pathogenesis suggest that amyloid and tau‐related neurodegeneration occur as separate but sequential pathologic events where amyloid beta drives the pathogenesis of Alzheimer disease and tau neuropathology occurs secondarily and closer to the onset of progressive cognitive decline (Kametani & Hasegawa, 2018; Leuzy et al., 2019). In line with this, mutation carriers have tau PET values similar to non‐carriers, as long as their cortical amyloid beta levels does not meet the threshold for positivity, suggesting that cortical amyloid beta facilitates tau pathology (Fleisher et al., 2012; Fleisher et al., 2015; Gordon et al., 2019; Quiroz et al., 2018). This model may partly explain two of the main findings in our study: (1) there is a significant relationship between tau deposition and image intensity metrics (mainly T1‐*σ*, FLAIR‐*σ*, and FLAIR‐*μ*) in mutation carriers specifically after symptomatic onset, compared with the lack of a similar relationship with amyloid uptake; and (2) the relationships between these metrics and EYO uptake are exponential in select cortical regions. These results persisted after accounting for regional atrophy, a notable finding given considerable cortical thinning in these regions within the mutation carrier group (Benzinger et al., 2013; Jack et al., 2020). Similarly, the pattern and timing of onset of tau deposition in mutation carriers, resembles that of the differences observed in T1‐*σ*, FLAIR‐*σ*, and FLAIR‐*μ* between mutation carriers and non‐carriers. Given that these abnormalities are detected after accounting for atrophy and given that cortical atrophy in DIAN does not become apparent until at least 5 years before symptoms onset (Benzinger et al., 2013), our findings suggests a prominent role for tau pathology independent from atrophy in the observed signal changes.

We used a well‐defined cohort of individuals with dominantly inherited Alzheimer disease with a predictable pathological time course and age of symptomatic onset, to study a novel intensity‐based MR biomarker derived from standard clinical images. We found that mutation carriers are distinguished from non‐carriers by differences in their image intensity metrics and there is a clear relationship between our novel MR metrics and tau neuropathology. Our MR metrics are readily applicable to standard T1w and FLAIR images and we suggest that they could be used as a screening tool for tau pathology in this patient cohort. The efficacy of this processing pipeline can be further improved by reducing the number of quantified brain regions to as little as a single region of interest. The scalability of these quantifications is enabled by the large number of MRI examinations in the United States alone, at 39 million (Ladapo et al., 2018).

## FUNDING INFORMATION

DIAN was established in 2008 with the grant U19AG032438 to Washington University (JC Morris, PI) from the National Institute on Aging. The imaging core lab is supported by the following grants; NIA/NINDA U01AG042791‐02 Phase II, NIH/NIA UF1AG032438, NIH/NIA U19AG03243808 and the German Center for Neurodegenerative Diseases (DZNE) as well as the NIH/NIA R01AG052550‐01A1 for Tau PET. Generous support also was provided by grants from an anonymous foundation and from the philanthropy of F Simmons and O Mohan. To date, this project continues to be supported by the National Institute on Aging (DIAN, U19AG032438), the German Center for Neurodegenerative Diseases (DZNE), Raul Carrea Institute for Neurological Research (FLENI), the Research and Development Grants for Dementia from Japan Agency for Medical Research and Development (AMED), and the Korea Health Technology R&D Project through the Korea Health Industry Development Institute (KHIDI), and the Korea Dementia Research Center (KDRC), funded by the Korean Ministry of Health & Welfare and Ministry of Science & ICT.

## CONFLICT OF INTEREST STATEMENT

MRB has received compensation for consulting from Genentech, EMD Serono, and the Department of Justice. TLSB has investigator‐initiated research funding from the NIH, the Alzheimer's Association, the Barnes‐Jewish Hospital Foundation and Avid Radiopharmaceuticals (a wholly‐owned subsidiary of Eli Lilly). Dr. Benzinger participates as a site investigator in clinical trials sponsored by Avid Radiopharmaceuticals, Eli Lilly, Biogen, Eisai, Jansen, and Roche, an unpaid consultant to Eisai and Siemens, and is on an Advisory Board and Speaker's Bureau for Biogen. GSD's research is supported by NIH (K23AG064029, U01AG057195, U01NS120901, U19AG032438), the Alzheimer's Association, and Chan Zuckerberg Initiative. He serves as a consultant for Parabon Nanolabs Inc, as a Topic Editor (Dementia) for DynaMed (EBSCO), and as the Clinical Director of the Anti‐NMDA Receptor Encephalitis Foundation (Inc, Canada; uncompensated). He is the co‐Project PI for a clinical trial in anti‐NMDAR encephalitis, which receives support from Horizon Pharmaceuticals. He has developed educational materials for PeerView Media, Inc, and Continuing Education Inc. He owns stock in ANI pharmaceuticals. Dr. Day's institution has received support from Eli Lilly for Dr. Day's development and participation in an educational event promoting early diagnosis of symptomatic Alzheimer disease. PS holds research funding from the Australian National Health and Medical Research Council and Medical Research Future Fund. JL reports speaker fees from Bayer Vital, Biogen, EISAI, TEVA and Roche, consulting fees from Axon Neuroscience and Biogen, author fees from Thieme medical publishers and W. Kohlhammer GmbH medical publishers and is inventor in a patent “Oral Phenylbutyrate for Treatment of Human 4‐Repeat Tauopathies” (EP 23156122.6) filed by LMU Munich. In addition, he reports compensation for serving as chief medical officer for MODAG GmbH, is beneficiary of the phantom share program of MODAG GmbH and is inventor in a patent “Pharmaceutical Composition and Methods of Use” (EP 22159408.8) filed by MODAG GmbH, all activities outside the submitted work. DMC is supported by the UK Dementia Research Institute which receives its funding from DRI Ltd, funded by the UK Medical Research Council, Alzheimer's Society and Alzheimer's Research UK (ARUK‐PG2017‐1946), the UCL/UCLH NIHR Biomedical Research Centre, and the UKRI Innovation Scholars: Data Science Training in Health and Bioscience (MR/V03863X/1). GE Healthcare holds a license agreement with the University of Pittsburgh based on the [^11^C]PiB PET technology described in this manuscript. Drs. Klunk and Mathis are co‐inventors of [^11^C]PiB and, as such, have a financial interest in this license agreement. GE Healthcare provided no grant support for this study and had no role in the design or interpretation of results or preparation of this manuscript. All other authors have no conflicts of interest with this work and had full access to all of the data in the study and take responsibility for the integrity of the data and the accuracy of the data analysis.

## Supporting information


**Figure S1.** Surface demonstration of FreeSurfer‐based cortical regions with significant partial correlation between regional PIB and tau uptake and image intensity metrics using mutation carrier participants only.

**Figure S2.** Surface demonstration of FreeSurfer‐based cortical regions with significant partial correlation between regional PIB and tau uptake and image intensity metrics symptomatic mutation carriers only.

**Figure S3.** Comparison of absolute correlation coefficient values between tau and amyloid uptake in mutation carriers (left) and symptomatic mutation carriers (right).

**Data S1.** Supporting Information.

## Data Availability

DIAN‐Obs data that was used in this study can be accessed upon review and approval of both the pertinent DIAN Core leaders and the Principal Investigator overseeing the grant (https://dian.wustl.edu/our-research/for-investigators/dian-observational-study-investigator-resources/data-and-biospecimens-available-for-request/).
