# Supplementary figures and images for: T1 and FLAIR signal intensities are related to tau pathology in dominantly inherited Alzheimer disease
Source: Hum Brain Mapp. 2023 Oct 23;44(18):6375–87. doi: 10.1002/hbm.26514 (PMC10681640; doi:10.1002/hbm.26514)

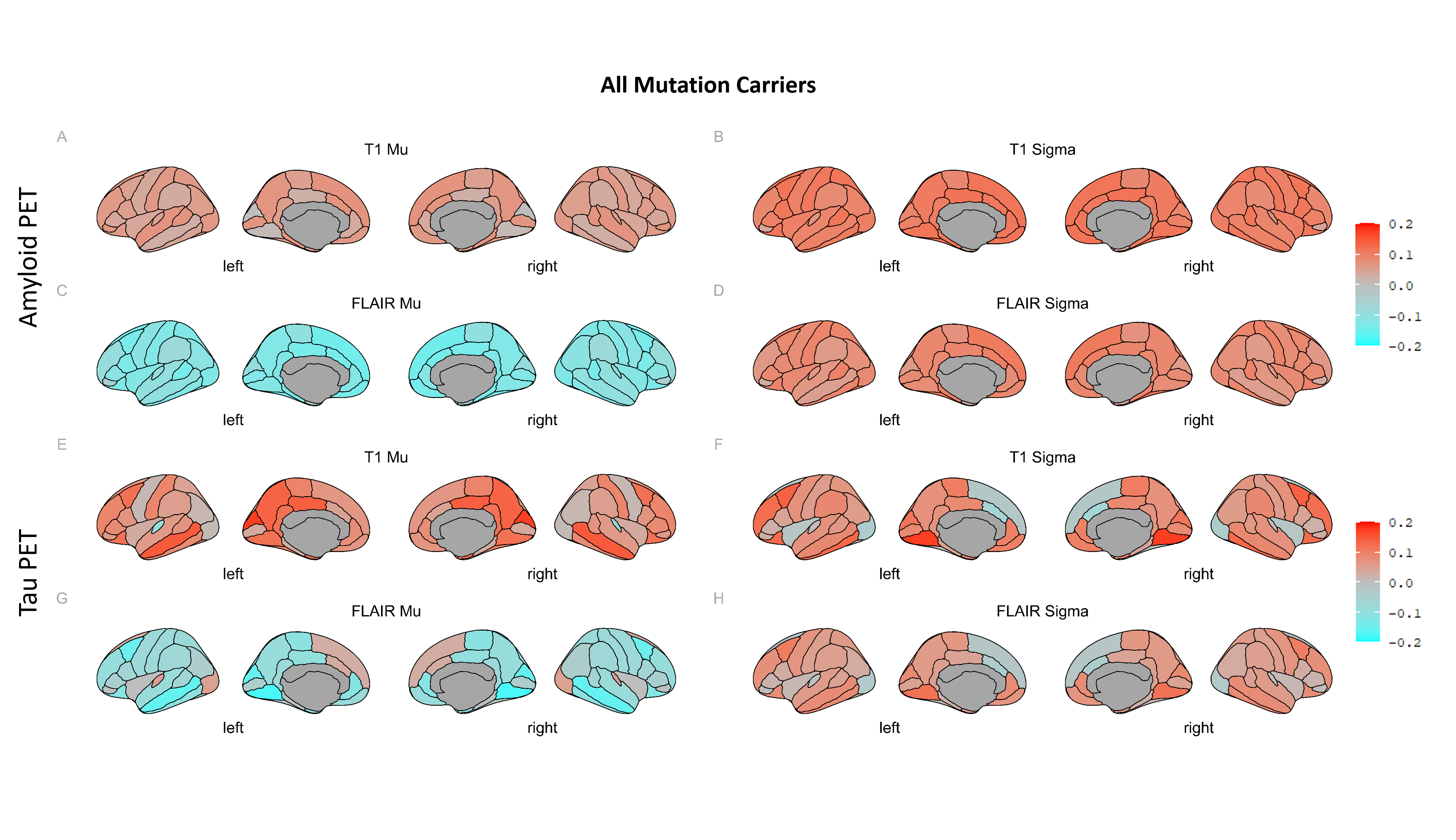

Supplement: Supplementary file 1 — Figure S1. Surface demonstration of FreeSurfer‐based cortical regions with significant partial correlation between regional PIB and tau uptake and image intensity metrics using mutation carrier participants only. [file HBM-44-6375-s002.png]

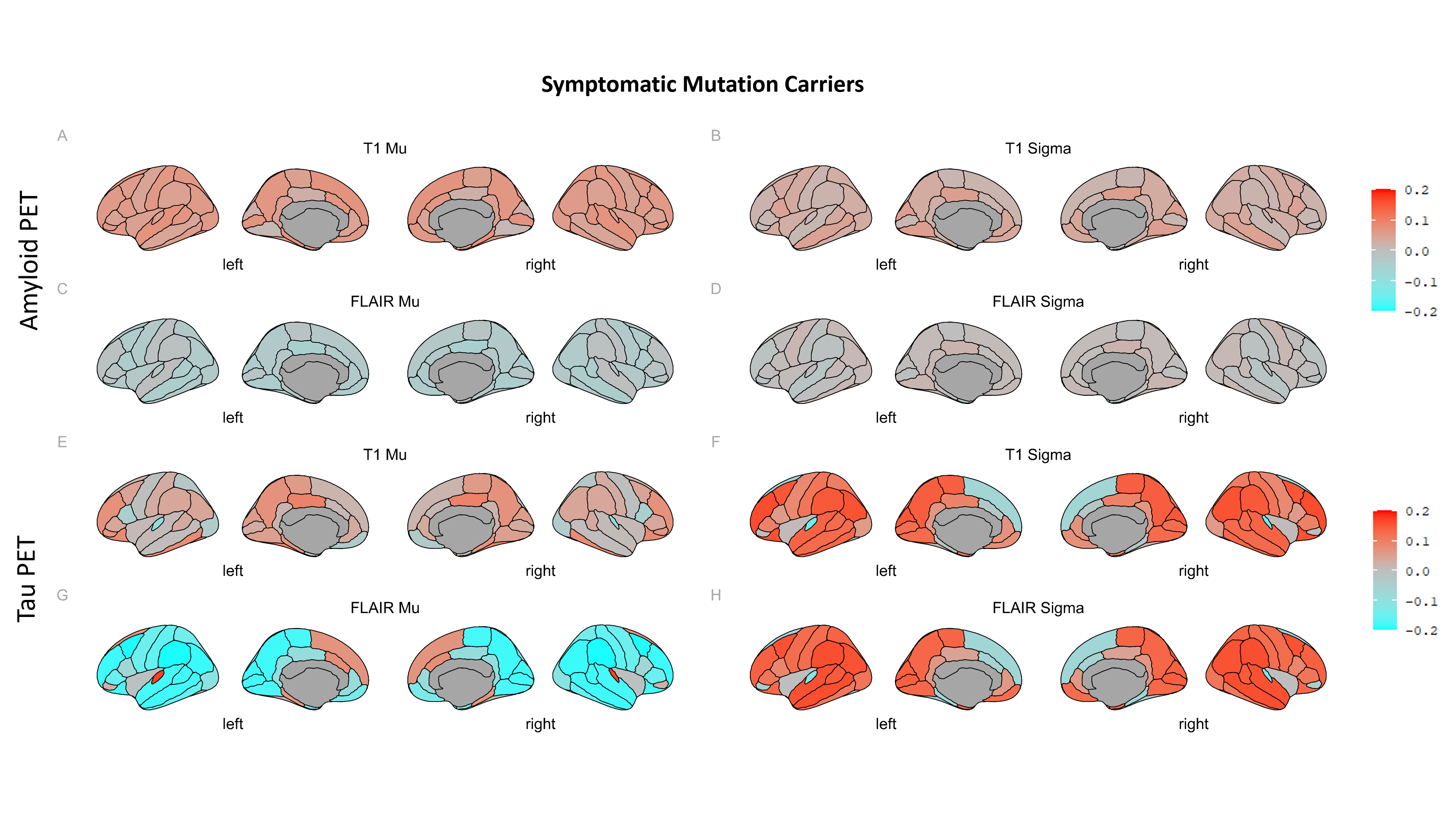

Supplement: Supplementary file 2 — Figure S2. Surface demonstration of FreeSurfer‐based cortical regions with significant partial correlation between regional PIB and tau uptake and image intensity metrics symptomatic mutation carriers only. [file HBM-44-6375-s003.png]

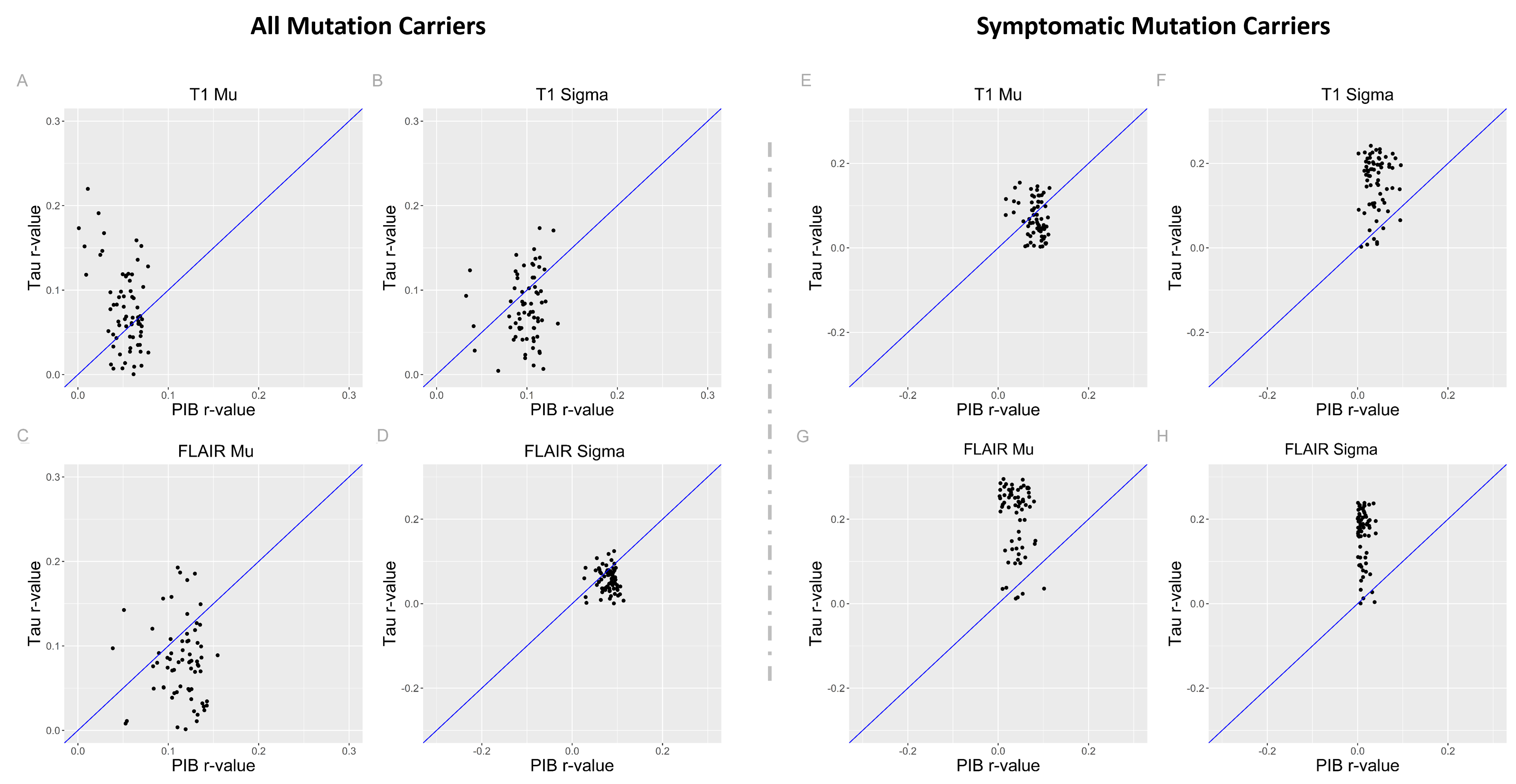

Supplement: Supplementary file 3 — Figure S3. Comparison of absolute correlation coefficient values between tau and amyloid uptake in mutation carriers (left) and symptomatic mutation carriers (right). [file HBM-44-6375-s001.png]
