# Supplementary material for: T1 and FLAIR signal intensities are related to tau pathology in dominantly inherited Alzheimer disease
Source: Hum Brain Mapp. 2023 Oct 23;44(18):6375–87. doi: 10.1002/hbm.26514 (PMC10681640; doi:10.1002/hbm.26514)
Supplement: Supplementary file 4 — Data S1. Supporting Information. [file HBM-44-6375-s004.docx]

| Supplementary Table 1. Participants and session characteristics contributing to each set of longitudinal analyses | | | | | |
| --- | --- | --- | --- | --- | --- |
| Longitudinal scan type | Total included visits (n) | Number of participants (n) | Between visit gap, month (mean±SD) | Number of visits (median(min-max)) | Total person-time (months) |
| MR (T1/FLAIR) | 1104 | 525 | 42.03±25.1 | 2 (1-8) | 14998 |
| Amyloid PET | 324 | 290 | 30.6±16.8 | 1 (1-2) | 9202 |
| Tau PET | 87 | 55 | 53.1±25.1 | 1 (1-4) | 2143 |
| Abbreviations:  *PET: positron imaging tomography; Amyloid PET: [11C]-Pittsburgh compound B (PiB)-based amyloid imaging; Tau PET: ^18^F-AV-1451 (flortaucipir) based tau imaging* | | | | | |

**Supplementary Table 2.** P-values of baseline and longitudinal model terms to predict the average T1 signal (T1-μ) in FreeSurfer-based cortical regions

| **Regions** | **Using Baseline Data points** | | | | | **Using Longitudinal Data points** | | | | |
| --- | --- | --- | --- | --- | --- | --- | --- | --- | --- | --- |
|  | **Hemi** | **EYO×Mut^*^** | **ANOVA****^*^** | **Time×EYO×Mut****^§^** | **ANOVA^§^** | **Hemi** | **EYO×Mut^*^** | **ANOVA^*^** | **Time×EYO×Mut^§^** | **ANOVA^§^** |
| Banks Of Superior Temporal Sulcus | Left | 0.11 | 0.78 | 0.64 | 0.71 | Right | 0.092 | 0.91 | 0.16 | 0.70 |
| Caudal Anterior Cingulate | Left | 0.79 | 0.56 | 0.63 | 0.18 | Right | 0.65 | 0.73 | 0.83 | 0.32 |
| Caudal Middle Frontal | Left | 0.53 | 0.92 | 0.53 | 0.81 | Right | 0.26 | 0.83 | 0.66 | 0.68 |
| Cuneus | Left | 0.11 | 0.96 | ***0.027*** | 0.80 | Right | 0.081 | 0.73 | ***0.025*** | 0.85 |
| Entorhinal | Left | 0.34 | 0.58 | 0.082 | 0.49 | Right | 0.17 | 0.73 | 0.67 | 0.63 |
| Fusiform | Left | 0.05 | 0.73 | 0.21 | 0.63 | Right | ***0.045*** | 0.77 | 0.82 | 0.68 |
| Inferior Parietal | Left | 0.92 | 0.92 | 0.18 | 0.85 | Right | 0.140 | 0.73 | 0.54 | 0.71 |
| Inferior Temporal | Left | 0.25 | 0.78 | ***0.048*** | 0.70 | Right | ***0.045*** | 0.73 | 0.36 | 0.49 |
| Isthmus Cingulate | Left | ***0.045*** | 0.92 | 0.54 | 0.70 | Right | 0.18 | 0.56 | 0.57 | 0.18 |
| Lateral Occipital | Left | 0.11 | 0.96 | 0.83 | 0.90 | Right | 0.11 | 0.96 | 0.53 | 0.73 |
| Lateral Orbitofrontal | Left | 0.5 | 0.27 | 0.16 | 0.18 | Right | 0.65 | 0.56 | 0.18 | 0.26 |
| Lingual | Left | 0.3 | 0.73 | ***0.044*** | 0.49 | Right | 0.17 | 0.73 | 0.22 | 0.27 |
| Medial Orbitofrontal | Left | 0.48 | 0.56 | 0.36 | 0.27 | Right | 0.29 | 0.56 | 0.24 | 0.27 |
| Middle Temporal | Left | 0.09 | 0.58 | 0.06 | 0.27 | Right | 0.12 | 0.92 | 0.64 | 0.71 |
| Parahippocampal | Left | 0.92 | 0.73 | 0.69 | 0.33 | Right | 0.30 | 0.73 | 0.81 | 0.27 |
| Paracentral | Left | 0.07 | 0.73 | 0.51 | 0.81 | Right | 0.37 | 0.73 | 0.77 | 0.76 |
| Pars Opercularis | Left | 0.65 | 0.73 | 0.83 | 0.49 | Right | 0.34 | 0.92 | 0.71 | 0.49 |
| Pars Orbitalis | Left | 0.34 | 0.56 | 0.24 | 0.27 | Right | 0.19 | 0.56 | 0.20 | 0.27 |
| Pars Triangularis | Left | 0.91 | 0.72 | 0.97 | 0.43 | Right | 0.91 | 0.91 | 0.16 | 0.71 |
| Pericalcarine | Left | 0.12 | 0.96 | 0.29 | 0.71 | Right | 0.09 | 0.99 | 0.21 | 0.80 |
| Postcentral | Left | 0.86 | 0.92 | 0.062 | 0.80 | Right | 0.69 | 0.73 | 0.90 | 0.80 |
| Posterior Cingulate | Left | 0.11 | 0.96 | 0.204 | 0.70 | Right | 0.65 | 0.96 | 0.91 | 0.70 |
| Precentral | Left | ***0.007*** | 0.73 | 0.95 | 0.71 | Right | 0.34 | 0.73 | 0.28 | 0.71 |
| Precuneus | Left | ***0.005*** | 0.73 | 0.43 | 0.71 | Right | ***0.005*** | 0.73 | 0.87 | 0.85 |
| Rostral Anterior Cingulate | Left | 0.89 | 0.73 | 0.52 | 0.27 | Right | 0.39 | 0.58 | 0.94 | 0.27 |
| Rostral Middle Frontal | Left | 0.019 | 0.06 | 0.52 | ***0.04*** | Right | ***0.045*** | 0.11 | 0.30 | 0.18 |
| Superior Frontal | Left | 0.7 | 0.73 | 0.86 | 0.49 | Right | 0.92 | 0.73 | 0.62 | 0.49 |
| Superior Parietal | Left | 0.7 | 0.96 | 0.19 | 0.82 | Right | 0.11 | 0.73 | 0.74 | 0.80 |
| Superior Temporal | Left | 0.058 | 0.92 | 0.82 | 0.80 | Right | 0.17 | 0.99 | 0.53 | 0.81 |
| Supramarginal | Left | 0.65 | 0.92 | 0.54 | 0.82 | Right | 0.12 | 0.73 | 0.25 | 0.70 |
| Frontal Pole | Left | 0.051 | 0.56 | 0.72 | 0.28 | Right | 0.051 | 0.58 | 0.21 | 0.27 |
| Temporal Pole | Left | 0.78 | 0.92 | ***0.025*** | 0.71 | Right | 0.46 | 0.73 | 0.18 | 0.70 |
| Transverse Temporal | Left | 0.18 | 0.92 | 0.19 | 0.63 | Right | 0.81 | 0.73 | 0.21 | 0.73 |
| Insula | Left | 0.39 | 0.58 | 0.23 | 0.27 | Right | 0.68 | 0.73 | 0.69 | 0.49 |
| Amygdala | Left | 0.20 | 0.88 | 0.99 | 0.11 | Right | 0.15 | 0.08 | 0.51 | 0.40 |
| Caudate | Left | 0.20 | 0.38 | 0.34 | 0.73 | Right | 0.20 | 0.19 | 0.47 | 0.40 |
| Hippocampus | Left | 0.84 | 0.33 | 0.34 | 0.11 | Right | 0.84 | 0.81 | 0.15 | 0.12 |
| Pallidum | Left | 0.88 | 0.10 | 0.09 | 0.72 | Right | 0.80 | 0.63 | 0.23 | ***0.038*** |
| Putamen | Left | 0.20 | 0.17 | 0.20 | 0.96 | Right | 0.15 | 0.39 | 0.47 | 0.39 |
| Thalamus | Left | 0.99 | 0.10 | 0.99 | 0.92 | Right | 0.92 | 0.41 | 0.52 | 0.12 |
| ** P-values of the interaction term between DIAN-EYO×Mutation from Model 1 and the ANOVA test to compare Model 1 and Model 3 using baseline data points only.*  *§ P-values of the interaction term between Time×EYO×Mutation from Model 2 and the ANOVA test to compare Model 2 and Model 4 using all longitudinal data points.* *Bold text represents significant effects considering a p-value threshold of 0.05.*  *Abbreviations: Hemi: hemisphere; Mut: mutation* | | | | | | | | | | |

**Supplementary Table 3.** P-values of baseline and longitudinal model terms to predict the T1 signal variability (T1-σ) in FreeSurfer-based cortical regions

| **Regions** | **Using Baseline Data points** | | | | | **Using Longitudinal Data points** | | | | |
| --- | --- | --- | --- | --- | --- | --- | --- | --- | --- | --- |
|  | **Hemi** | **EYO×Mut^*^** | **ANOVA^*^** | **Time×EYO×Mut^§^** | **ANOVA^§^** | **Hemi** | **EYO×Mut^*^** | **ANOVA^*^** | **Time×EYO×Mut^§^** | **ANOVA^§^** |
| Banks Of Superior Temporal Sulcus | Left | ***<0.001*** | ***0.001*** | 0.21 | ***<0.001*** | Right | ***<0.001*** | ***0.009*** | 0.10 | ***<0.001*** |
| Caudal Anterior Cingulate | Left | ***0.033*** | 0.98 | 0.11 | 0.339 | Right | 0.015 | 0.81 | 0.18 | 0.433 |
| Caudal Middle Frontal | Left | ***<0.001*** | ***0.010*** | 0.7 | ***0.001*** | Right | ***<0.001*** | ***0.001*** | ***0.038*** | ***<0.001*** |
| Cuneus | Left | ***<0.001*** | ***0.015*** | 0.06 | ***0.008*** | Right | 0.016 | 0.45 | ***0.019*** | 0.339 |
| Entorhinal | Left | ***0.003*** | ***0.009*** | 0.19 | ***<0.001*** | Right | ***<0.001*** | 0.48 | 0.15 | 0.294 |
| Fusiform | Left | ***0.026*** | 0.064 | 0.90 | ***0.001*** | Right | 0.087 | 0.06 | 0.23 | ***0.005*** |
| Inferior Parietal | Left | ***<0.001*** | ***0.005*** | 0.46 | ***0.001*** | Right | ***<0.001*** | ***0.035*** | 0.60 | ***0.002*** |
| Inferior Temporal | Left | 0.085 | 0.32 | 0.84 | ***0.004*** | Right | 0.089 | ***0.043*** | 0.56 | ***0.001*** |
| Isthmus Cingulate | Left | ***<0.001*** | ***0.009*** | ***0.003*** | ***<0.001*** | Right | ***<0.001*** | ***0.001*** | ***0.020*** | ***<0.001*** |
| Lateral Occipital | Left | ***0.003*** | 0.06 | 0.42 | ***0.004*** | Right | ***<0.001*** | ***0.024*** | 0.75 | ***0.003*** |
| Lateral Orbitofrontal | Left | ***0.026*** | 0.12 | ***0.047*** | 0.198 | Right | 0.363 | 0.33 | 0.40 | 0.381 |
| Lingual | Left | ***<0.001*** | 0.10 | ***0.021*** | ***0.013*** | Right | ***<0.001*** | ***0.001*** | ***0.054*** | ***<0.001*** |
| Medial Orbitofrontal | Left | ***0.015*** | 0.46 | 0.72 | 0.402 | Right | 0.029 | 0.34 | 0.20 | 0.101 |
| Middle Temporal | Left | ***0.003*** | 0.13 | 0.42 | ***0.002*** | Right | ***<0.001*** | ***0.043*** | 0.38 | ***<0.001*** |
| Parahippocampal | Left | ***0.003*** | ***0.013*** | ***0.018*** | ***0.001*** | Right | 0.949 | ***0.002*** | ***0.003*** | ***<0.001*** |
| Paracentral | Left | ***0.018*** | 0.11 | 0.11 | ***0.008*** | Right | 0.055 | 0.17 | 0.87 | ***0.043*** |
| Pars Opercularis | Left | ***<0.001*** | ***0.001*** | 0.12 | ***<0.001*** | Right | ***<0.01*** | ***0.001*** | 0.52 | ***<0.001*** |
| Pars Orbitalis | Left | ***0.006*** | ***0.003*** | 0.44 | ***0.001*** | Right | ***0.013*** | 0.29 | 0.76 | 0.101 |
| Pars Triangularis | Left | ***0.001*** | ***0.012*** | 0.83 | ***<0.001*** | Right | ***0.001*** | 0.07 | 0.57 | ***0.008*** |
| Pericalcarine | Left | ***<0.001*** | ***0.009*** | 0.54 | ***0.002*** | Right | ***0.001*** | ***0.049*** | 0.07 | ***0.008*** |
| Postcentral | Left | ***<0.001*** | ***0.003*** | ***0.004*** | ***0.001*** | Right | ***0.001*** | ***0.001*** | 0.91 | ***<0.001*** |
| Posterior Cingulate | Left | ***<0.001*** | ***0.009*** | ***0.016*** | ***0.001*** | Right | ***<0.001*** | ***0.033*** | 0.15 | ***<0.001*** |
| Precentral | Left | 0.37 | 0.20 | 0.09 | ***0.009*** | Right | ***0.028*** | ***0.042*** | 0.80 | ***0.003*** |
| Precuneus | Left | ***<0.001*** | 0.05 | 0.12 | ***<0.001*** | Right | ***<0.001*** | ***0.013*** | ***0.003*** | ***<0.001*** |
| Rostral Anterior Cingulate | Left | ***0.007*** | 0.48 | 0.39 | ***0.040*** | Right | ***0.013*** | 0.253 | 0.10 | 0.429 |
| Rostral Middle Frontal | Left | ***<0.001*** | ***<0.001*** | 0.13 | ***<0.001*** | Right | ***<0.001*** | ***0.001*** | ***0.031*** | **0.001** |
| Superior Frontal | Left | ***<0.001*** | ***0.003*** | ***0.040*** | ***<0.001*** | Right | ***<0.001*** | ***0.001*** | 0.08 | ***<0.001*** |
| Superior Parietal | Left | ***<0.001*** | ***0.001*** | 0.63 | ***<0.001*** | Right | ***<0.001*** | ***0.007*** | ***0.007*** | ***<0.001*** |
| Superior Temporal | Left | ***0.036*** | ***0.003*** | 0.16 | ***<0.001*** | Right | ***0.005*** | ***0.018*** | 0.58 | ***<0.001*** |
| Supramarginal | Left | ***<0.001*** | ***0.002*** | 0.06 | ***<0.001*** | Right | ***0.003*** | ***0.006*** | 0.11 | ***<0.001*** |
| Frontal Pole | Left | 0.33 | 0.54 | 0.40 | 0.294 | Right | 0.158 | 0.17 | 0.88 | 0.088 |
| Temporal Pole | Left | 0.08 | 0.33 | 0.44 | ***0.039*** | Right | 0.319 | 0.98 | 0.61 | 0.951 |
| Transverse Temporal | Left | ***0.008*** | ***0.010*** | 0.10 | ***<0.001*** | Right | 0.141 | 0.05 | 0.19 | ***0.002*** |
| Insula | Left | ***0.001*** | 0.452 | 0.65 | ***0.033*** | Right | ***0.03*** | 0.18 | 0.52 | ***0.016*** |
| Amygdala | Left | 0.91 | 0.82 | 0.70 | 0.54 | Right | 0.30 | 0.46 | 0.57 | 0.57 |
| Caudate | Left | 0.42 | 0.51 | 0.06 | 0.45 | Right | 0.36 | 0.31 | 0.45 | 0.45 |
| Hippocampus | Left | 0.21 | 0.46 | 0.19 | 0.27 | Right | 0.39 | 0.46 | 0.37 | 0.37 |
| Pallidum | Left | 0.26 | 0.07 | 0.90 | 0.89 | Right | 0.40 | 0.46 | 0.57 | 0.57 |
| Putamen | Left | ***<0.001*** | 0.07 | ***0.02*** | ***0.033*** | Right | ***<0.01*** | 0.07 | 0.90 | 0.90 |
| Thalamus | Left | 0.30 | 0.51 | 0.12 | 0.89 | Right | 0.30 | 0.9 | 0.99 | 0.99 |
| ** P-value of the interaction term between DIAN-EYO×Mutation from Model 1 and the ANOVA test to compare Model 1 and Model 3 using baseline data points only.*  *§ P-value of the interaction term between Time×EYO×Mutation from Model 2 and the ANOVA test to compare Model 2 and Model 4 using all longitudinal data points.*  *Bold text represents significant effects considering a p-value threshold of 0.05.*  *Abbreviations: Hemi: hemisphere; Mut: mutation* | | | | | | | | | | |

**Supplementary Table 4.** P-values of baseline and longitudinal model terms to predict the average FLAIR signal (FLAIR-µ) in FreeSurfer-based cortical regions

| **Regions** | **Using Baseline Data points** | | | | | **Using Longitudinal Data points** | | | | |
| --- | --- | --- | --- | --- | --- | --- | --- | --- | --- | --- |
|  | **Hemi** | **EYO×Mut^*^** | **ANOVA^*^** | **Time×EYO×Mut^§^** | **ANOVA^§^** | **Hemi** | **EYO×Mut^*^** | **ANOVA^*^** | **Time×EYO×Mut^§^** | **ANOVA^§^** |
| Banks Of Superior Temporal Sulcus | Left | ***<0.001*** | ***0.021*** | ***0.005*** | ***0.001*** | Right | ***0.001*** | 0.08 | ***0.001*** | ***0.002*** |
| Caudal Anterior Cingulate | Left | 0.064 | ***0.015*** | ***0.023*** | ***0.006*** | Right | ***0.027*** | ***0.015*** | 0.10 | ***0.003*** |
| Caudal Middle Frontal | Left | ***<0.001*** | ***0.024*** | 0.16 | ***0.009*** | Right | ***0.001*** | ***0.020*** | ***0.008*** | ***0.007*** |
| Cuneus | Left | ***<0.001*** | ***<0.001*** | ***0.005*** | ***<0.001*** | Right | ***0.001*** | ***0.002*** | ***0.008*** | ***0.001*** |
| Entorhinal | Left | 0.162 | 0.08 | ***<0.001*** | ***0.027*** | Right | ***0.014*** | 0.06 | 0.068 | ***0.008*** |
| Fusiform | Left | 0.107 | 0.06 | ***<0.001*** | ***0.014*** | Right | ***0.041*** | ***0.002*** | ***<0.001*** | ***<0.001*** |
| Inferior Parietal | Left | ***<0.001*** | ***0.004*** | ***0.001*** | ***<0.001*** | Right | ***<0.001*** | ***0.002*** | ***0.005*** | ***<0.001*** |
| Inferior Temporal | Left | ***0.017*** | 0.05 | 0.09 | ***0.005*** | Right | 0.252 | 0.50 | ***<0.001*** | ***0.024*** |
| Isthmus Cingulate | Left | 0.086 | **0.024** | ***0.007*** | ***0.001*** | Right | 0.248 | ***0.020*** | 0.10 | ***0.005*** |
| Lateral Occipital | Left | ***0.006*** | 0.24 | 0.56 | ***0.010*** | Right | **0.001** | ***0.023*** | 0.65 | ***0.003*** |
| Lateral Orbitofrontal | Left | ***0.005*** | ***0.027*** | 0.85 | ***0.010*** | Right | **0.023** | 0.26 | 0.38 | ***0.041*** |
| Lingual | Left | ***<0.001*** | ***0.010*** | ***0.001*** | ***<0.001*** | Right | 0.204 | 0.05 | 0.15 | ***0.003*** |
| Medial Orbitofrontal | Left | **0.002** | 0.14 | 0.194 | ***0.044*** | Right | **0.002** | ***0.044*** | 0.55 | ***0.008*** |
| Middle Temporal | Left | ***<0.001*** | ***0.004*** | 0.20 | ***<0.001*** | Right | ***<0.001*** | ***0.002*** | ***0.001*** | ***<0.001*** |
| Parahippocampal | Left | 0.16 | 0.14 | 0.19 | ***0.010*** | Right | 0.830 | 0.191 | 0.20 | ***0.026*** |
| Paracentral | Left | ***0.007*** | 0.20 | ***0.008*** | 0.09 | Right | ***0.006*** | 0.130 | 0.67 | 0.122 |
| Pars Opercularis | Left | ***<0.001*** | ***0.002*** | ***<0.001*** | ***<0.001*** | Right | ***<0.001*** | ***0.001*** | ***<0.001*** | ***<0.001*** |
| Pars Orbitalis | Left | ***0.011*** | ***0.020*** | 0.73 | ***0.006*** | Right | ***0.004*** | 0.25 | 0.87 | 0.122 |
| Pars Triangularis | Left | ***<0.001*** | 0.14 | 0.90 | ***0.019*** | Right | ***0.033*** | 0.08 | 0.06 | ***0.024*** |
| Pericalcarine | Left | ***<0.001*** | ***<0.001*** | 0.05 | ***<0.001*** | Right | ***0.003*** | ***<0.001*** | 0.27 | ***<0.001*** |
| Postcentral | Left | **0.002** | ***0.003*** | ***0.001*** | ***0.002*** | Right | ***0.006*** | ***<0.001*** | ***0.028*** | ***<0.001*** |
| Posterior Cingulate | Left | ***<0.001*** | ***0.003*** | ***0.038*** | ***0.001*** | Right | ***0.005*** | ***0.004*** | ***0.027*** | ***0.007*** |
| Precentral | Left | 0.13 | 0.26 | 0.10 | 0.07 | Right | ***0.034*** | 0.15 | ***0.010*** | ***0.038*** |
| Precuneus | Left | ***<0.001*** | ***<0.001*** | ***<0.001*** | ***<0.001*** | Right | ***<0.001*** | ***0.003*** | ***<0.001*** | ***<0.001*** |
| Rostral Anterior Cingulate | Left | ***<0.001*** | ***0.004*** | ***0.009*** | ***0.001*** | Right | ***<0.001*** | ***<0.001*** | ***0.006*** | ***<0.001*** |
| Rostral Middle Frontal | Left | ***<0.001*** | ***<0.001*** | 0.206 | ***<0.001*** | Right | ***<0.001*** | ***0.001*** | ***0.025*** | ***0.001*** |
| Superior Frontal | Left | ***<0.001*** | 0.072 | ***0.039*** | ***0.014*** | Right | ***<0.001*** | ***0.049*** | 0.062 | ***0.010*** |
| Superior Parietal | Left | ***<0.001*** | ***0.021*** | 0.126 | ***<0.001*** | Right | ***<0.001*** | 0.08 | ***0.004*** | ***0.001*** |
| Superior Temporal | Left | ***<0.001*** | ***0.003*** | ***0.005*** | ***<0.001*** | Right | ***<0.001*** | ***<0.001*** | ***0.001*** | ***<0.001*** |
| Supramarginal | Left | ***<0.001*** | ***<0.001*** | ***<0.001*** | ***<0.001*** | Right | ***<0.001*** | ***<0.001*** | ***<0.001*** | ***<0.001*** |
| Frontal Pole | Left | ***0.002*** | 0.21 | 0.97 | 0.13 | Right | ***0.002*** | 0.28 | 0.47 | 0.118 |
| Temporal Pole | Left | ***0.008*** | 0.078 | 0.20 | ***0.008*** | Right | ***0.011*** | 0.065 | 0.75 | ***0.021*** |
| Transverse Temporal | Left | ***<0.001*** | ***<0.001*** | ***<0.001*** | ***<0.001*** | Right | ***0.001*** | ***<0.001*** | ***<0.001*** | ***<0.001*** |
| Insula | Left | ***<0.001*** | ***0.002*** | ***0.014*** | ***<0.001*** | Right | ***<0.001*** | ***0.002*** | 0.075 | ***0.001*** |
| Amygdala | Left | 0.08 | 0.20 | 0.62 | 0.29 | Right | ***<0.01*** | 0.24 | ***<0.001*** | ***0.023*** |
| Caudate | Left | ***<0.01*** | 0.80 | 0.09 | 0.29 | Right | ***0.011*** | 0.94 | ***<0.01*** | 0.36 |
| Hippocampus | Left | 0.11 | 0.22 | 0.19 | 0.07 | Right | 0.24 | 0.83 | 0.40 | 0.46 |
| Pallidum | Left | 0.08 | 0.20 | 0.19 | ***0.012*** | Right | ***<0.01*** | 0.46 | ***<0.001*** | 0.09 |
| Putamen | Left | ***0.026*** | 0.80 | 0.05 | 0.31 | Right | ***<0.01*** | 0.23 | ***<0.001*** | 0.36 |
| Thalamus | Left | ***<0.001*** | 0.94 | ***<0.01*** | ***<0.01*** | Right | ***<0.001*** | 0.23 | ***<0.001*** | 0.11 |
| ** P-value of the interaction term between DIAN-EYO×Mutation from Model 1 and the ANOVA test to compare Model 1 and Model 3 using baseline data points only.*  *§ P-value of the interaction term between Time×EYO×Mutation from Model 2 and the ANOVA test to compare Model 2 and Model 4 using all longitudinal data points.*  *Bold text represents significant effects considering a p-value threshold of 0.05.*  *Abbreviations: Hemi: hemisphere; Mut: mutation* | | | | | | | | | | |

**Supplementary Table 5.** P-values of baseline and longitudinal model terms to predict the variability in FLAIR signal (FLAIR-σ) in FreeSurfer-based cortical regions

| **Regions** | **Using Baseline Data points** | | | | | **Using Longitudinal Data points** | | | | |
| --- | --- | --- | --- | --- | --- | --- | --- | --- | --- | --- |
|  | **Hemi** | **EYO×Mut^*^** | **ANOVA^*^** | **Time×EYO×Mut^§^** | **ANOVA^§^** | **Hemi** | **EYO×Mut^*^** | **ANOVA^*^** | **Time×EYO×Mut^§^** | **ANOVA^§^** |
| Banks Of Superior Temporal Sulcus | Left | ***0.005*** | ***<0.01*** | ***0.006*** | 0.001 | Right | ***0.005*** | ***0.003*** | ***<0.001*** | ***0.006*** |
| Caudal Anterior Cingulate | Left | ***0.040*** | 0.25 | 0.51 | 0.34 | Right | 0.075 | ***0.014*** | 0.55 | 0.512 |
| Caudal Middle Frontal | Left | ***0.001*** | ***<0.01*** | 0.52 | ***<0.01*** | Right | ***0.005*** | ***0.004*** | ***0.010*** | 0.521 |
| Cuneus | Left | ***0.001*** | ***0.035*** | ***0.011*** | ***0.02*** | Right | 0.071 | 0.05 | ***0.007*** | ***0.011*** |
| Entorhinal | Left | 0.972 | 0.67 | 0.56 | 0.55 | Right | 0.487 | 0.92 | 0.98 | 0.565 |
| Fusiform | Left | 0.972 | 0.73 | 0.53 | 0.62 | Right | 0.818 | 0.54 | 0.13 | 0.533 |
| Inferior Parietal | Left | ***<0.001*** | 0.06 | 0.41 | 0.07 | Right | ***<0.001*** | 0.11 | 0.55 | 0.414 |
| Inferior Temporal | Left | 0.82 | 0.37 | 0.42 | 0.34 | Right | 0.913 | 0.84 | 0.76 | 0.420 |
| Isthmus Cingulate | Left | 0.81 | ***0.02*** | 0.70 | ***<0.01*** | Right | 0.818 | 0.07 | 0.95 | 0.709 |
| Lateral Occipital | Left | 0.37 | 0.42 | 0.90 | 0.23 | Right | ***0.007*** | 0.32 | 0.99 | 0.907 |
| Lateral Orbitofrontal | Left | ***0.045*** | ***0.03*** | 0.10 | ***0.03*** | Right | 0.380 | 0.17 | 0.26 | 0.108 |
| Lingual | Left | ***0.016*** | 0.92 | 0.29 | 0.62 | Right | 0.801 | 0.26 | 0.81 | 0.296 |
| Medial Orbitofrontal | Left | ***0.034*** | 0.49 | 0.49 | 0.16 | Right | ***0.034*** | 0.16 | 0.19 | 0.499 |
| Middle Temporal | Left | 0.82 | 0.58 | 0.96 | 0.47 | Right | 0.191 | 0.83 | 0.35 | 0.968 |
| Parahippocampal | Left | 0.82 | 0.92 | 0.18 | 0.92 | Right | 0.82 | 0.92 | 0.85 | 0.187 |
| Paracentral | Left | 0.26 | 0.07 | 0.05 | 0.013 | Right | ***0.039*** | 0.54 | 0.91 | 0.054 |
| Pars Opercularis | Left | ***0.045*** | 0.04 | ***0.027*** | 0.013 | Right | ***0.025*** | ***0.035*** | 0.60 | ***0.027*** |
| Pars Orbitalis | Left | 0.19 | 0.07 | 0.65 | ***0.041*** | Right | 0.06 | 0.34 | 0.96 | 0.659 |
| Pars Triangularis | Left | ***0.014*** | 0.26 | 0.89 | 0.094 | Right | ***0.048*** | 0.34 | 0.78 | 0.893 |
| Pericalcarine | Left | ***<0.001*** | ***<0.01*** | ***<0.001*** | ***<0.001*** | Right | ***0.001*** | 0.13 | ***0.027*** | ***<0.001*** |
| Postcentral | Left | ***0.007*** | ***0.015*** | ***0.025*** | ***<0.01*** | Right | ***0.024*** | ***<0.001*** | 0.58 | ***0.025*** |
| Posterior Cingulate | Left | ***0.001*** | ***<0.01*** | ***0.002*** | ***0.001*** | Right | ***0.004*** | 0.001 | ***0.008*** | ***0.002*** |
| Precentral | Left | 0.67 | 0.61 | 0.072 | 0.352 | Right | ***0.025*** | 0.263 | 0.19 | 0.072 |
| Precuneus | Left | ***0.005*** | ***0.01*** | ***0.003*** | ***<0.001*** | Right | ***0.042*** | ***0.009*** | ***0.004*** | ***0.003*** |
| Rostral Anterior Cingulate | Left | ***0.001*** | ***0.03*** | 0.41 | ***<0.01*** | Right | ***<0.001*** | ***0.016*** | 0.53 | 0.410 |
| Rostral Middle Frontal | Left | ***0.001*** | ***<0.01*** | 0.28 | ***0.022*** | Right | ***0.010*** | ***0.008*** | 0.07 | 0.284 |
| Superior Frontal | Left | ***0.001*** | ***0.037*** | 0.09 | ***<0.01*** | Right | ***0.001*** | ***0.023*** | 0.52 | 0.099 |
| Superior Parietal | Left | ***<0.001*** | ***0.01*** | 0.93 | ***0.001*** | Right | ***<0.001*** | 0.11 | ***0.011*** | 0.932 |
| Superior Temporal | Left | 0.80 | 0.14 | 0.15 | ***0.049*** | Right | 0.12 | 0.07 | 0.66 | 0.158 |
| Supramarginal | Left | ***<0.001*** | ***<0.001*** | 0.16 | ***<0.001*** | Right | ***0.005*** | ***<0.001*** | 0.06 | 0.165 |
| Frontal Pole | Left | 0.81 | 0.95 | 0.40 | 0.92 | Right | 0.82 | 0.36 | 0.87 | 0.406 |
| Temporal Pole | Left | 0.66 | 0.42 | 0.71 | 0.22 | Right | 0.81 | 0.87 | 0.88 | 0.71 |
| Transverse Temporal | Left | ***0.001*** | 0.076 | ***<0.001*** | ***0.02*** | Right | ***0.024*** | ***0.010*** | 0.07 | ***<0.001*** |
| Insula | Left | ***<0.01*** | ***0.012*** | 0.624 | ***0.001*** | Right | ***0.001*** | ***0.011*** | 0.37 | 0.624 |
| Amygdala | Left | ***<0.001*** | ***0.031*** | ***<0.01*** | ***<0.01*** | Right | 0.45 | 0.06 | 0.61 | 0.68 |
| Caudate | Left | 0.45 | 0.51 | 0.66 | ***0.033*** | Right | 0.45 | 0.52 | 0.61 | 0.90 |
| Hippocampus | Left | ***<0.001*** | 0.6 | ***<0.001*** | ***<0.01*** | Right | 0.45 | 0.25 | 0.62 | 0.90 |
| Pallidum | Left | 0.45 | 0.6 | 0.66 | 0.76 | Right | 0.67 | 0.52 | 0.80 | 0.072 |
| Putamen | Left | 0.48 | 0.06 | 0.06 | ***0.038*** | Right | 0.81 | 0.41 | ***0.022*** | ***<0.01*** |
| Thalamus | Left | ***<0.001*** | ***0.03*** | ***<0.01*** | ***<0.001*** | Right | ***0.045*** | 0.52 | ***0.041*** | ***<0.01*** |
| ** P-value of the interaction term between DIAN-EYO×Mutation from Model 1 and the ANOVA test to compare Model 1 and Model 3 using baseline data points only.*  *§ P-value of the interaction term between Time×EYO×Mutation from Model 2 and the ANOVA test to compare Model 2 and Model 4 using all longitudinal data points.*  *Bold text represents significant effects considering a p-value threshold of 0.05.*  *Abbreviations: Hemi: hemisphere; Mut: mutation* | | | | | | | | | | |

**
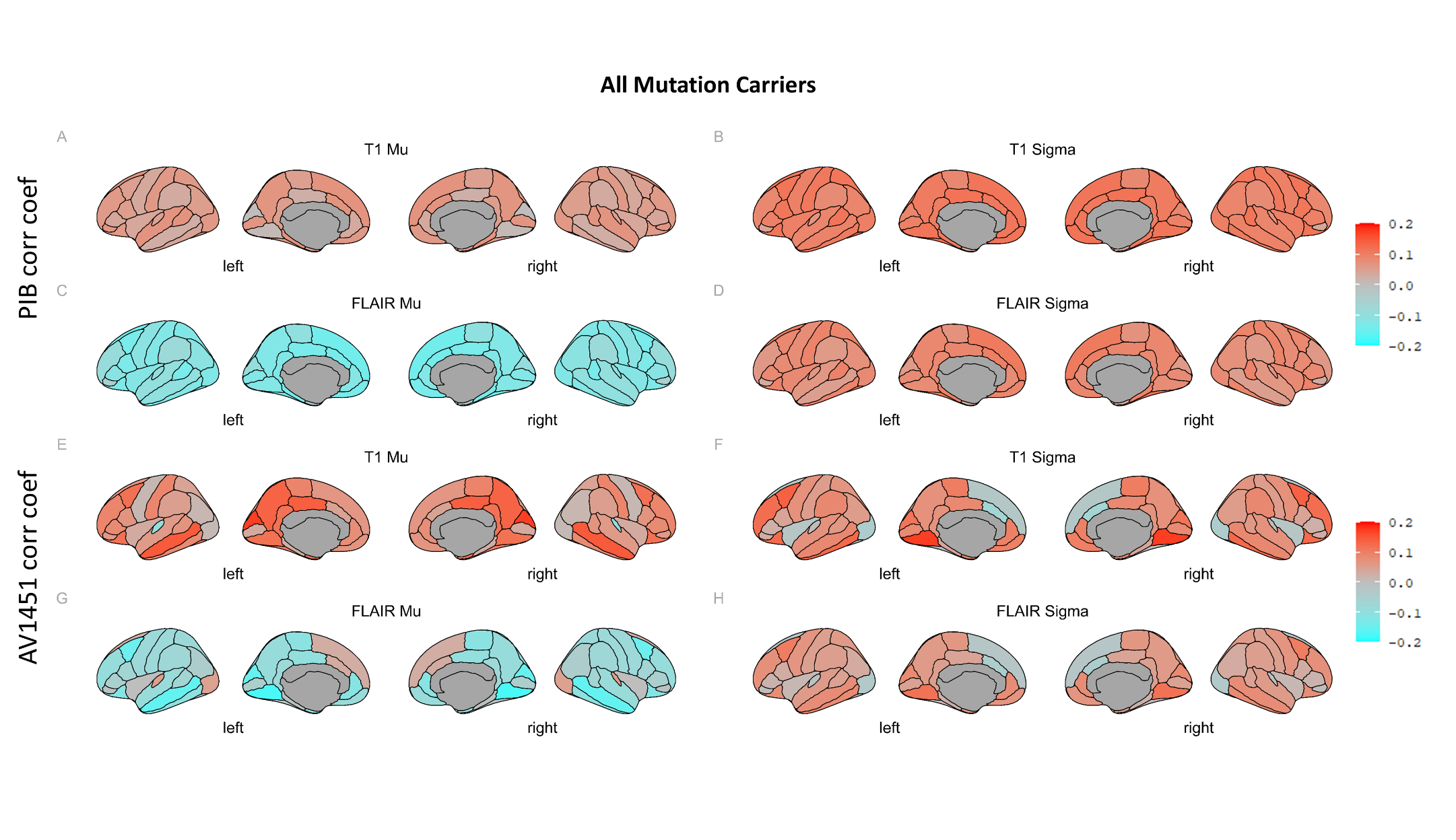
Supplementary Figure 1** Surface demonstration of FreeSurfer-based cortical regions with significant partial correlation between regional PIB and tau uptake and image intensity metrics using mutation carrier participants only

*Panels A through D demonstrate the negative log10 of p-value for the Pearson’s correlation test between regional amyloid burden and image intensity metrics after controlling for the effect of regional cortical volumes. As a result the intensity of the colors are proportional to the p-value of the partial correlation whereby lower p-values result in higher color intensity in that region. Note that all regions disregarding the FDR-corrected p-values significance are demonstrated.*

*Panels E through H demonstrate the negative log10 of p-value for the Pearson’s correlation test between regional amyloid burden and image intensity metrics after controlling for the effect of regional cortical volumes. As a result the intensity of the colors are proportional to the p-value of the partial correlation whereby lower p-values result in higher color intensity in that region. Note that all regions disregarding the FDR-corrected p-values significance are demonstrated.*


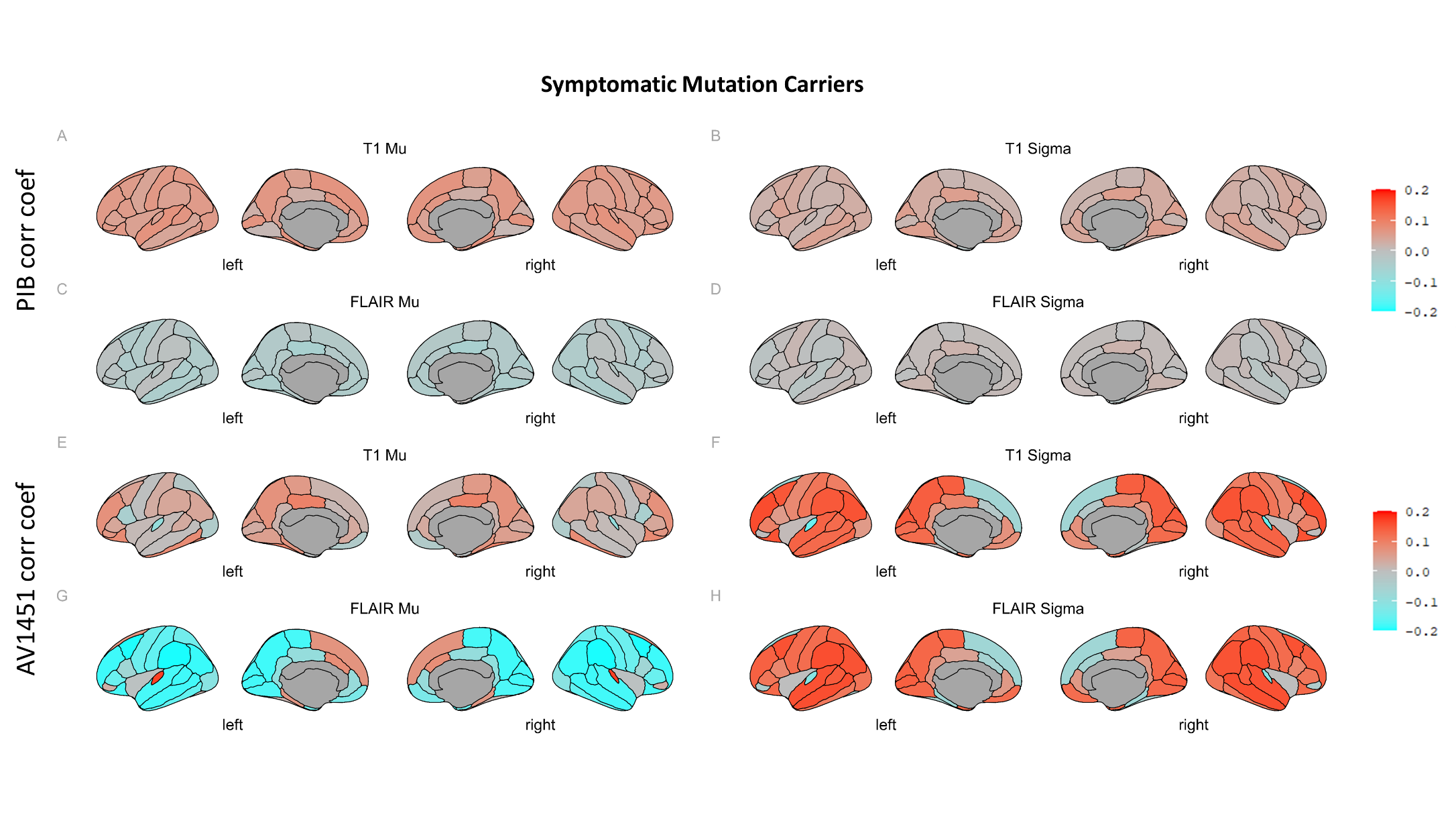
**Supplementary Figure 2.** Surface demonstration of FreeSurfer-based cortical regions with significant partial correlation between regional PIB and tau uptake and image intensity metrics symptomatic mutation carriers only

*Panels A through D demonstrate the negative log10 of p-value for the Pearson’s correlation test between regional amyloid burden and image intensity metrics after controlling for the effect of regional cortical volumes. As a result the intensity of the colors are proportional to the p-value of the partial correlation whereby lower p-values result in higher color intensity in that region. Note that all regions disregarding the FDR-corrected p-values significance are demonstrated.*

*Panels E through H demonstrate the negative log10 of p-value for the Pearson’s correlation test between regional amyloid burden and image intensity metrics after controlling for the effect of regional cortical volumes. As a result the intensity of the colors are proportional to the p-value of the partial correlation whereby lower p-values result in higher color intensity in that region. Note that all regions disregarding the FDR-corrected p-values significance are demonstrated.*


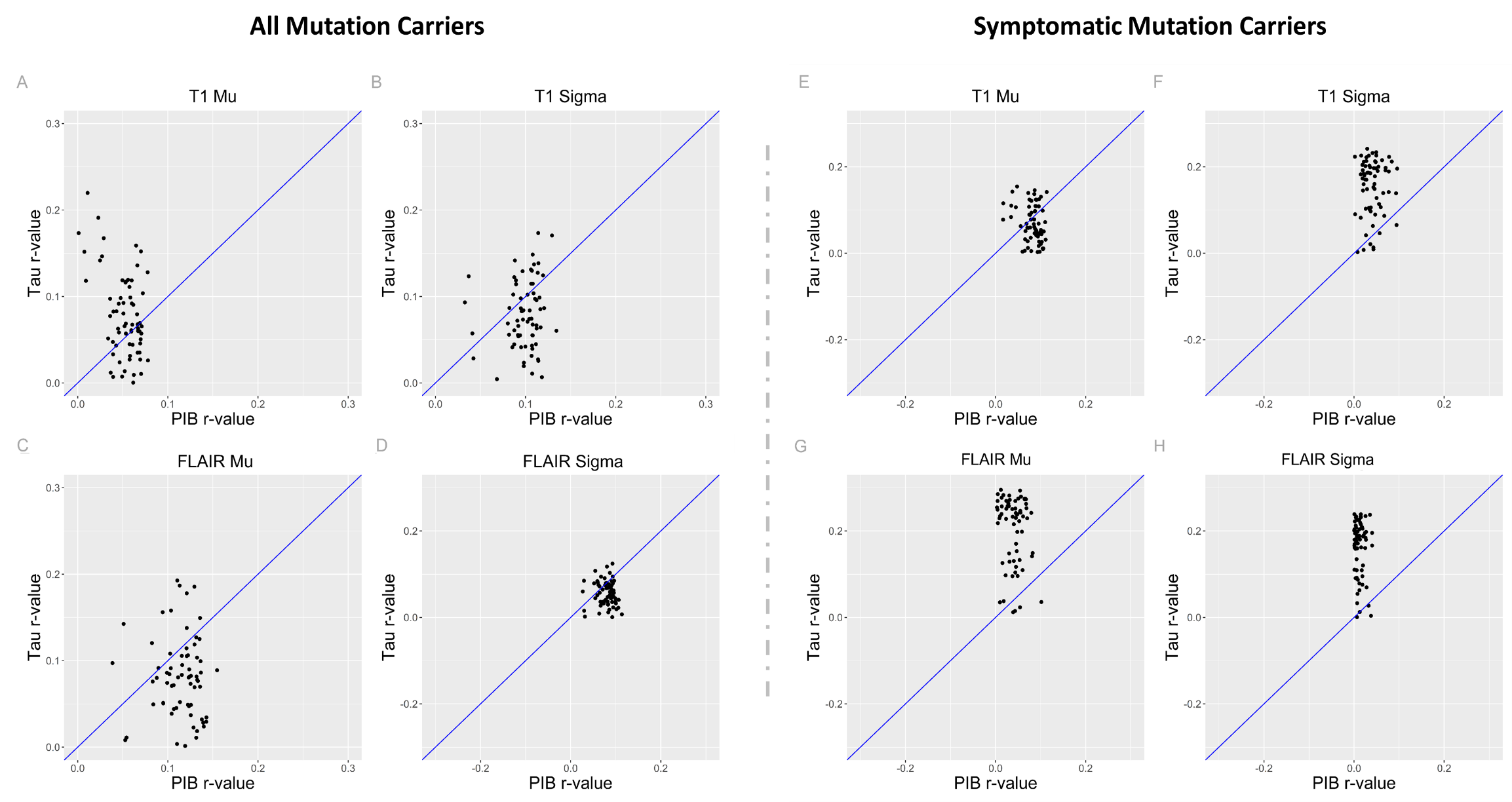
**Supplementary Figure 3.** Comparison of absolute correlation coefficient values between tau and amyloid uptake in mutation carriers (left) and symptomatic mutation carriers (right)

*X-axis demonstrates absolute correlation coefficients of image intensity metrics and PIB uptake, while the Y-axis demonstrates absolute correlation coefficients of image intensity metrics and tau uptake. Each black dot represents one cortical region. The blue line denotes the X=Y regression line. r-value: correlation coefficient*
